# Energy Metabolism Regulates Stem Cell Pluripotency

**DOI:** 10.3389/fcell.2020.00087

**Published:** 2020-02-28

**Authors:** Enkhtuul Tsogtbaatar, Chelsea Landin, Katherine Minter-Dykhouse, Clifford D. L. Folmes

**Affiliations:** Stem Cell and Regenerative Metabolism Laboratory, Departments of Cardiovascular Diseases and Biochemistry and Molecular Biology, Mayo Clinic, Scottsdale, AZ, United States

**Keywords:** naïve and primed embryonic stem cells, induced pluripotent stem cells, nuclear reprogramming, glycolysis, oxidative metabolism, oxidative phosphorylation, tricarboxylic acid cycle, amino acids

## Abstract

Pluripotent stem cells (PSCs) are characterized by their unique capacity for both unlimited self-renewal and their potential to differentiate to all cell lineages contained within the three primary germ layers. While once considered a distinct cellular state, it is becoming clear that pluripotency is in fact a continuum of cellular states, all capable of self-renewal and differentiation, yet with distinct metabolic, mitochondrial and epigenetic features dependent on gestational stage. In this review we focus on two of the most clearly defined states: “naïve” and “primed” PSCs. Like other rapidly dividing cells, PSCs have a high demand for anabolic precursors necessary to replicate their genome, cytoplasm and organelles, while concurrently consuming energy in the form of ATP. This requirement for both anabolic and catabolic processes sufficient to supply a highly adapted cell cycle in the context of reduced oxygen availability, distinguishes PSCs from their differentiated progeny. During early embryogenesis PSCs adapt their substrate preference to match the bioenergetic requirements of each specific developmental stage. This is reflected in different mitochondrial morphologies, membrane potentials, electron transport chain (ETC) compositions, and utilization of glycolysis. Additionally, metabolites produced in PSCs can directly influence epigenetic and transcriptional programs, which in turn can affect self-renewal characteristics. Thus, our understanding of the role of metabolism in PSC fate has expanded from anabolism and catabolism to include governance of the pluripotent epigenetic landscape. Understanding the roles of metabolism and the factors influencing metabolic pathways in naïve and primed pluripotent states provide a platform for understanding the drivers of cell fate during development. This review highlights the roles of the major metabolic pathways in the acquisition and maintenance of the different states of pluripotency.

## Pluripotent Stem Cell Fate *in utero* and *in vitro*

Pluripotency describes the developmental capacity of a cell to form the three primary germ layers, which in turn can give rise to all cell types of the adult body. As such it does not represent a single fixed stage, but rather a gradient of cellular phenotypes from the “naïve” pluripotent stem cells (PSCs) of the inner cell mass (ICM) of the early blastocyst, to the “primed” PSCs of the early post-implantation epiblast (Weinberger et al., 2016). Upon uterine implantation, the epiblast cells progressively lose expression of core pluripotency genes Oct4 and Nanog as the three germ layers emerge during gastrulation (Weinberger et al., 2016; Mathieu and Ruohola-Baker, 2017). The expression of these transcription factors is spatially restricted and ultimately suppressed by the time of somitogenesis (Osorno et al., 2012).

Although pluripotency exists transiently *in vivo*, several stages can be stably recapitulated *in vitro* (Evans and Kaufman, 1981). Embryonic stem cells (ESCs) derived from the ICM of murine blastocysts are considered the developmental naïve state in terms of their transcriptional activity, epigenetics and metabolic phenotypes (Nichols and Smith, 2009; Weinberger et al., 2016). General characteristics of naïve PSCs include the ability to give rise to all somatic lineages, incorporate into a developing blastocyst generating chimeric embryos, two active X chromosomes in female lines and the utilization of bivalent metabolism [both glycolysis and oxidative phosphorylation (OxPhos)] (Weinberger et al., 2016). While original protocols required leukemia inhibitory factor (LIF) and serum supplementation to maintain this naïve state, serum is dispensable upon the addition of GSK3 and MEK inhibitors (2i) (Ying et al., 2008). In contrast, when cells are derived from the post-implantation epiblast they are termed epiblast stem cells (mEpiSCs) and are considered a primed PSC, representative of a later developmental stage of pluripotency, and as such are functionally different from naïve PSCs (Brons et al., 2007; Tesar et al., 2007). Characteristics of primed PSCs do overlap with those of naïve PSCs, yet there are notable differences: primed PSCs express the core pluripotency genes Oct4, Sox2, and Nanog, however they are not capable of integrating into a developing blastocyst to form chimeric embryos, they are predominantly glycolytic, and inactivation of one X chromosome has been noted in female lines (Weinberger et al., 2016). Interestingly mEpiSCs can be cultured *in vitro* without LIF when in the presence of fibroblast growth factor (FGF) and activin A (Brons et al., 2007; Tesar et al., 2007).

**FIGURE 1:**
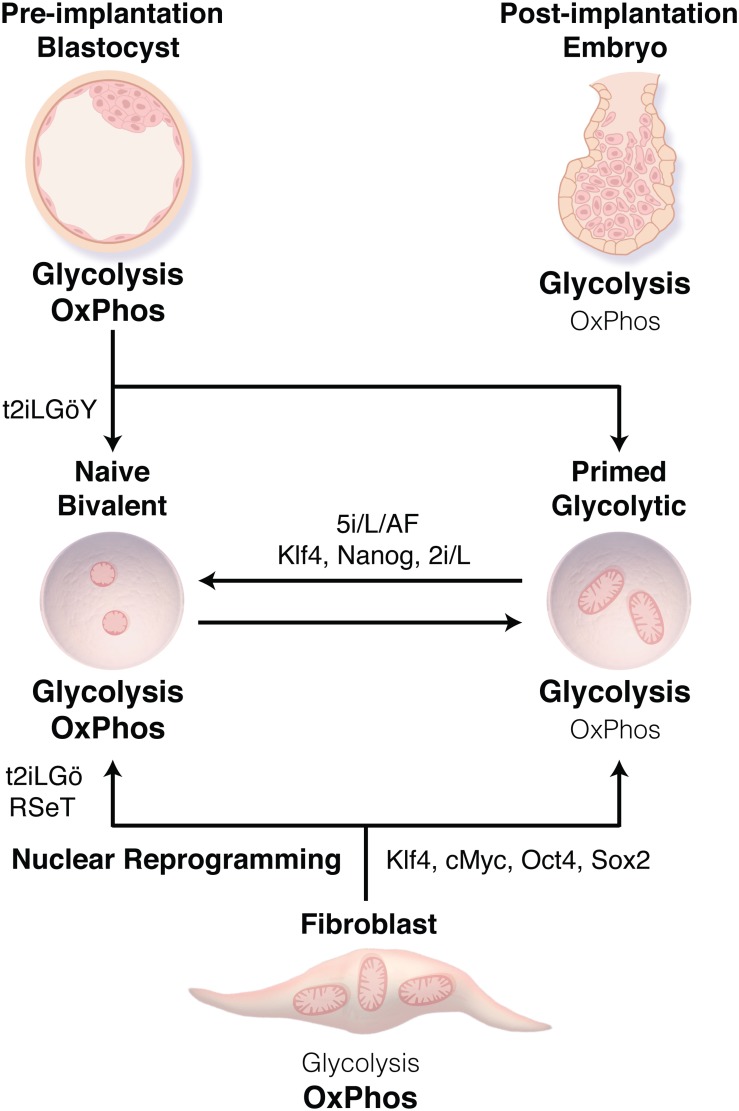
Transitions between human pluripotent stem cell (PSC) states. Human embryonic stem cells derived from the inner cell mass of the blastocyst or through nuclear reprogramming traditionally display a primed state associated with bivalent metabolism using both glycolysis and oxidative phosphorylation (OxPhos). Methods have now been developed to derive naïve PSCs (t2iLGö ± Y, RSeT) or to transition cells from the primed to naïve state (5i/L/AF, or Klf4, Nanog, 2i/L), resulting in a transition from bivalent metabolism to nearly exclusively glycolysis. These metabolic preferences reflect those of the related stage during embryonic development.

Unlike mESCs, human ESCs (hESCs) derived from the ICM of the human blastocyst resemble a primed rather than naïve state (Thomson et al., 1998) (Figure 1). This aligns hESCs more closely with mEpiSCs in terms of their culture requirements, as well as their transcriptional and epigenetic profiles. Reviews by Davidson et al. (2015) and Weinberger et al. (2016) discuss the growing body of literature highlighting the differences between naïve and primed ESCs from murine and human epiblasts in terms of transcriptomic, epigenetic and chromosomal profiles, and postulate these differences are likely the result of species specific developmental programs and requirements. A stable naïve state can be generated by culturing primed hESCs in a cocktail of MEK, RTK, BRAF, ROCK, and GSK3β inhibitors, in addition to LIF and activin A (5i/L/A) or titrated 2i with LIF and PKC inhibitor Gö6983 (t2iLGö) (Takashima et al., 2014; Theunissen et al., 2014). Similar conditions with the addition of ROCK inhibitor and ascorbic acid (t2iLGöY) have also been utilized to derive naïve ESCs from the human ICM (Guo et al., 2016). The resultant naïve hESCs recapitulate the features of mESCs, including X chromosome reactivation in female cell lines. Interestingly, inhibition of Rho kinase with the ROCK inhibitor Y-27632 in single cell hESCs dissociated by enzymatic methods initially results in the suppression of glycolysis, TCA cycle, and glutaminolysis, while promoting cell survival by inhibiting caspase-3 mediated apoptosis (Vernardis et al., 2017). Prolonged culture in ROCK inhibitor (>96 h) results in metabolic adaptation, after which hESC metabolism rebounds with both glycolysis and oxidative metabolism upregulated, an adaptation observed and attributed to the naïve hESC state. Whether the inclusion of ROCK inhibitor in media optimized for the generation of naïve hESCs functions primarily to couple metabolic flux with proliferation, or to inhibit apoptosis in the development of the naïve state is unclear.

Given ethical concerns and restrictions in deriving human ESC lines, combined with the fact that hESCs can only yield allogenic options for cell-based therapies; nuclear reprogramming has paved the way for the generation of patient derived induced pluripotent stem cells (iPSCs) (Takahashi and Yamanaka, 2006; Takahashi et al., 2007). This technology utilizes the exogenous expression of core pluripotency transcription factors OCT4, SOX2, KLF4, and c-MYC to reprogram somatic cells to a pluripotent state, producing human iPSCs comparable to primed human ESCs. In contrast, nuclear reprogramming of somatic murine cells results in pluripotency more reminiscent of naïve mESCs, which may be due to a better understanding of the mediators of the murine naïve pluripotent state, as it has recently been demonstrated that parallel isogenic primed and naïve iPSCs can be derived by using media supporting the naïve state, RSeT and T2iLGö (Kilens et al., 2018).

**FIGURE 2:**
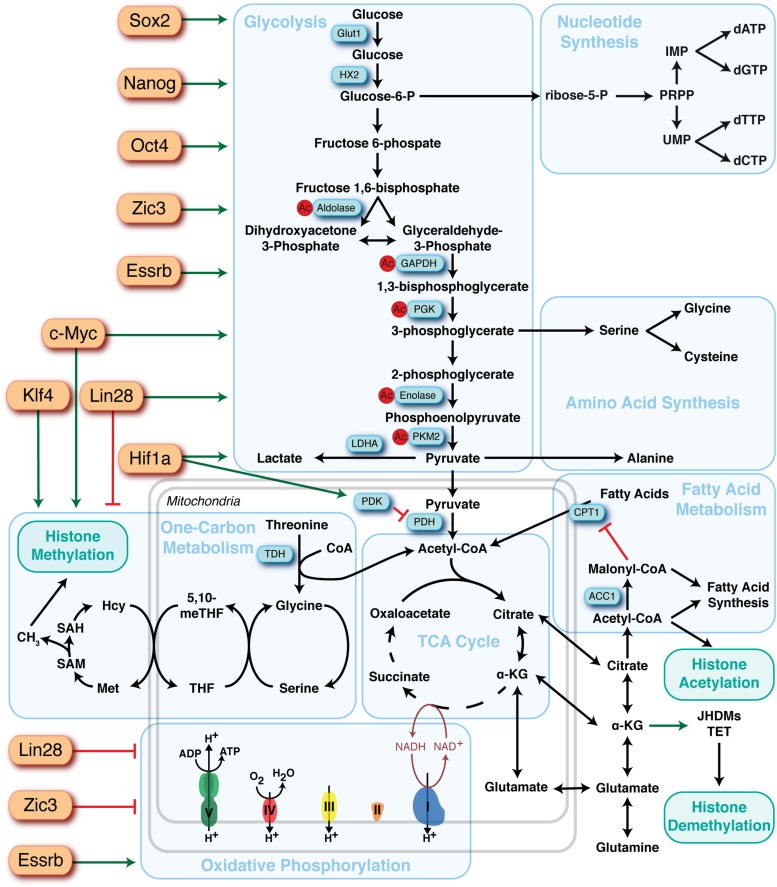
Overview of the major metabolic pathways in pluripotent stem cells and their regulators. Major metabolic pathways are found in blue boxes and key sites of regulation are highlighted in blue rectangles. Identified upstream regulators of metabolic pathways are depicted as orange rectangles and the directionality of regulation represented by red bars (suppression) or green arrows (activation).

**FIGURE 3:**
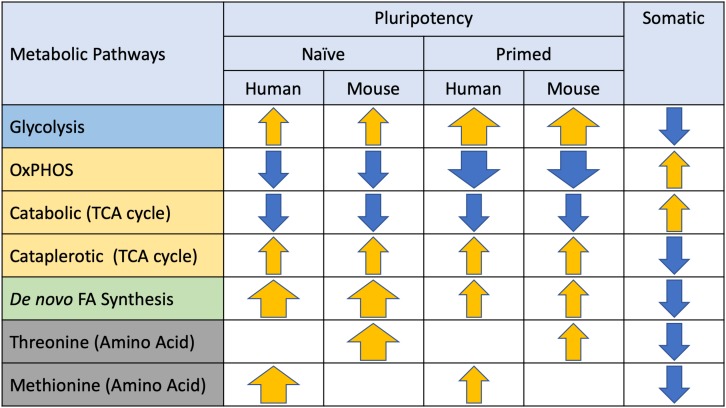
Metabolic preferences of naïve and primed pluripotent stem cells (PSCs). Major metabolic pathways identified in PSCs are listed on the left and arrows depicting directionality (yellow = elevated, blue = suppressed) and magnitude (thickness) of differences relative to somatic cells.

Distinct developmental stages are associated with specific patterns of metabolic activity, and growing evidence supports the concept that early metabolic changes are critical for regulating epigenetics and establishing cellular identity. Cells constantly modulate their metabolic state in response to nutrient availability, extracellular signals and reprogramming/differentiation cues, which induces changes in the concentrations of key intermediary metabolites that can serve as cofactors for the epigenetic regulation of gene transcription, including histone methylation and acetylation. This crosstalk between intermediary metabolism and epigenetics may in part account for how metabolic pathways can contribute to stem cell fate determination. In the context of pluripotency, studies have focused on several key metabolites, including S-adenosylmethionine (SAM), alpha-ketoglutarate (αKG) and acetyl-CoA, that sit at the nexus between intermediary metabolism and epigenetics. For example, methylation of discrete residues on histones play a fundamental role in regulating ESCs differentiation and pluripotent state. PSCs have chromatin characterized by “bivalent” domains, which are regulatory regions containing both an activating histone modification, histone H3 lysine 4 trimethylation (H3K4me3), and a repressive modification, histone H3 lysine 27 trimethylation (H3K27me3), which poise developmental genes to maintain their repression in the absence of differentiation signals, yet allow timely activation (Bernstein et al., 2006). Indeed, these critical histone marks for regulation of pluripotency and differentiation are in part regulated through metabolism (Kaelin and McKnight, 2013). H3K4me3 is dependent on SAM levels derived through one-carbon metabolism from exogenous or glucose-derived serine, threonine or methionine metabolism (Shyh-Chang et al., 2013b; Shiraki et al., 2014). Repressive H3K9me3 and H3K27me3 marks are regulated in an αKG-dependent manner through demethylation by JmjC-domain containing histone demethylases (JHDMs) and ten-eleven translocation (TET) enzymes (Kaelin and McKnight, 2013). H3K27 can also be acetylated, which marks enhancer regions and areas of elevated transcriptional activity. Acetylation is dependent on acetyl-CoA derived from glycolysis, which is critical for the regulation of pluripotency versus differentiation (Carey et al., 2015; TeSlaa et al., 2016; Tischler et al., 2019; Vardhana et al., 2019). The mechanisms by which metabolism regulates epigenetics have been reviewed extensively elsewhere (Ryall et al., 2015; Harvey et al., 2016a, 2019). This review focuses on the major metabolic pathways (Figure 2) that influence PSC identity and how they change, or can be modulated, to support acquisition of pluripotency through nuclear reprogramming and transition between the distinct stages of pluripotency (Figure 3).

## Pluripotent Stem Cell Metabolism

### Glycolysis

High glycolytic flux is common to various stem cell populations and is critical for the acquisition and maintenance of pluripotency (Ezashi et al., 2005; Cho et al., 2006; Kondoh et al., 2007; Varum et al., 2009, 2011; Simsek et al., 2010; Zhu et al., 2010; Folmes et al., 2011, 2012; Panopoulos et al., 2012; Takubo et al., 2013). Glycolysis is a sequence of cytosolic redox reactions that convert a single glucose molecule into two pyruvate molecules coupled with the generation of two net ATP and two reduced NADH molecules. While glycolysis is less energetically efficient compared to the complete oxidation of glucose in the tricarboxylic acid (TCA) cycle and OxPhos, this pathway can occur in the absence of oxygen and enables a fast rate of ATP production that can surpass that of oxygen-dependent respiration when glucose is abundant, as is the case in cell culture. In fact, ATP/ADP ratios seem to be unaffected by this metabolic preference in these cell types (Guppy et al., 1993). Indeed, highly proliferating cell types typically utilize glycolysis despite sufficient oxygen availability to support oxidative metabolism (aerobic glycolysis), as incomplete oxidation of glucose enables the conservation of carbon biomass required for biosynthesis of cellular components needed for daughter cell generation (Vander Heiden et al., 2009; Lunt and Vander Heiden, 2011). Glycolytic intermediates can also be shunted into different pathways to meet the high anabolic demands of proliferating cells: lipid synthesis via dihydroxyacetone phosphate and acetyl-CoA, and nucleotide and NADPH synthesis through glucose-6-phosphate and the Pentose Phosphate Pathway (PPP). The importance of the anabolic utilization of glycolytic intermediates is highlighted by ^13^C-labeling studies that confirm the utilization of glucose-derived carbons in the manufacturing of nucleotides and amino acids such as serine (Gu et al., 2016; Lees et al., 2019). The preferential utilization of glycolysis over mitochondrial oxidative metabolism may also represent a mechanism to preserve the genomic integrity of PSCs by reducing reactive oxygen species (ROS) produced by OxPhos and limiting subsequent damage of nuclear and mitochondrial DNA as well as reducing oxidation of proteins and lipids (Perales-Clemente et al., 2014).

Although glycolytic flux is common across all stages of pluripotency, the relative contributions of glycolysis versus OxPhos do vary. The primed pluripotent state of hPSCs and mEpiSCs is almost exclusively glycolytic (Tesar et al., 2007; Nichols and Smith, 2009; Takashima et al., 2014; Theunissen et al., 2014), whereas naïve hPSCs and mESCs utilize a bivalent metabolic system with a greater reliance on oxidative metabolism (Lee et al., 2012; Zhou et al., 2012; Si et al., 2013; Mu et al., 2015; Cha et al., 2017). Indeed, early preparatory phase glycolytic metabolites such as fructose 1, 6-bisphosphate are enriched in primed PSCs without accumulation of downstream metabolites, suggesting that these glycolytic intermediates may be consumed for anabolism or one-carbon metabolism (Tesar et al., 2007; Zhou et al., 2012; Gafni et al., 2013; Takashima et al., 2014; Sperber et al., 2015). In contrast, naïve hESCs exhibited both higher glucose and oxygen consumption, coupled with elevated lactate production compared to their primed counterparts (Gu et al., 2016). This suggests that naïve pluripotent cells may reside in a hypermetabolic state, however, further investigation is needed to understand the exact mechanisms and purpose behind this high energetic requirement in the naïve state (Gu et al., 2016). These metabolic preferences may reflect substrate availability and metabolic regulation during early embryogenesis, which has been reviewed extensively elsewhere (Gardner, 1998; Folmes et al., 2012; Shyh-Chang et al., 2013a; Folmes and Terzic, 2014; Gardner and Harvey, 2015; Zhang et al., 2018). The early embryo displays low glycolytic rates due the inhibition of hexokinase and phosphofructokinase 1 and is dependent on pyruvate supplied by ovarian follicle cells to support oxidative metabolism, with glycolysis progressively increasing during development due to higher glucose availability and glucose transporter expression (Barbehenn et al., 1978; Pantaleon and Kaye, 1998).

Core pluripotency factors have been demonstrated to directly regulate glycolysis in PSCs (Folmes et al., 2012; Zhang et al., 2012). OCT4 directly regulates transcription of hexokinase 2 and pyruvate kinase M2 (Kim et al., 2015), consistent with the preference of these isoforms in iPSCs (Folmes et al., 2011; Prigione et al., 2014; Qin et al., 2017). Recently a SOX2, OCT4, and NANOG binding site has been described at the GLUT1 enhancer, which appears to increase GLUT1 expression and downstream glycolytic flux in ESCs (Yu et al., 2019). Additionally, c-MYC has been identified as a strong driver of glycolysis in both the naïve and primed pluripotent states (Folmes et al., 2013b; Gu et al., 2016). Myc expression is regulated through a miR-290/371-MBD2 pathway, with miR-290/371 repressing MBD2 and leading to increased expression of Myc and its downstream transcriptional targets PKM2 and LDHA, supporting elevated glycolysis (Cao et al., 2015). The RNA binding protein LIN28a/b, which plays critical roles in embryonic development, acquisition, and maintenance of pluripotency, and the transition between naïve and primed pluripotency has been demonstrated to regulate glycolysis in addition to a number of metabolic pathways, including maintaining low mitochondrial function, one-carbon metabolism, and nucleotide metabolism (Shyh-Chang et al., 2013c; Zhang et al., 2016b).

A number of upstream regulators have been implicated in regulating the metabolic phenotype during the transition between the naïve and primed state. Early work demonstrated that HIF1α is sufficient to drive the glycolytic phenotype in the primed state through activin/nodal signaling (Zhou et al., 2012). More recently Ras has been implicated in controlling a number of events that are critical for the transition from naïve to primed pluripotency, including stimulation of glycolysis, epithelial-mesenchymal transition (marked by an increase in N-Cadherin) and an increase in H3K27me3. Interestingly, blocking glycolysis reversed the effect of Ras on N-Cadherin and H3K27me3, suggesting these effects are downstream of the metabolic remodeling (Altshuler et al., 2018). Recent work has demonstrated that downregulation of SIRT2 and upregulation of SIRT1 are a molecular signature of primed hPSCs, and that these NAD-dependent deacetylases regulate primed pluripotency through distinct mechanisms (Cha et al., 2017). The authors observed that miR-200c5p, an OCT4 induced miRNA, can directly downregulate SIRT2 mRNA and protein expression leading to hyper-acetylation of glycolytic enzymes (aldolase, glyceraldehyde-3-phosphate dehydrogenase, phosphoglycerate kinase, enolase, and pyruvate kinases) and acceleration of glycolysis. Knockdown of SIRT2 also suppressed OxPhos in fibroblasts by an unknown mechanism, enabling a higher efficiency of nuclear reprogramming. In contrast, SIRT1 expression is upregulated in primed pluripotency and inhibition of its upstream miRNA-34a, while increased SIRT1 expression promotes nuclear reprogramming (Lee et al., 2012; Cha et al., 2017). However, SIRT1 appears to elicit its downstream effects independent of metabolic regulation, potentially through the deacetylation of SOX2 and p53 leading to increased Nanog expression, and p21 inhibition (Lee et al., 2012; Si et al., 2013; Mu et al., 2015). miRNA-34a deficiency can also endow PSCs with features of totipotent blastomeres, suggesting that these pathways may also play an important role in restricting pluripotent developmental potential (Choi et al., 2017).

Downstream of glycolysis, the fate of pyruvate has been increasingly recognized to regulate stem cell function (Flores et al., 2017; Schell et al., 2017). Pyruvate represents the nexus of multiple metabolic pathways and can be metabolized to: (a) acetyl-CoA by pyruvate dehydrogenase (PDH) to enable entry and subsequent oxidation in the TCA cycle, (b) lactate by lactate dehydrogenase (LDH), in part to enable regeneration of NAD^+^ to support high glycolytic rates, and (c) oxaloacetate by pyruvate carboxylase to anapleurotically replenish TCA cycle intermediates that are being utilized for anabolic pathways. In PSCs, pyruvate is largely metabolized to lactate, with the remaining being catabolized in the TCA cycle, in contrast to differentiated cells where pyruvate is mainly catabolized. Multiple enzymes responsible for pyruvate flux into the TCA cycle are differentially regulated in naïve *versus* primed PSCs, such as pyruvate dehydrogenase kinase (PDK) (Zhou et al., 2012). For example, in primed PSCs, transport of pyruvate to the mitochondria is tightly regulated by HIF1α-induced expression of PDK 1-3, which phosphorylates and inhibits PDH, thus pushing pyruvate to lactate production (Mathieu and Ruohola-Baker, 2017). It was also found that UCP2, an ATP uncoupling protein, blocks the uptake of pyruvate into the TCA cycle (Zhang et al., 2011). Interestingly, a recent study demonstrated that supplementing hESC media with exogenous pyruvate enhanced OxPhos while suppressing glycolysis, but had no impact on expression of pluripotent markers such as *Nanog, Pou5f1*, and *Sox2*, although this metabolic shift supported mesodermal differentiation through activation of the AMPK/mTOR pathway (Song et al., 2019). In summary, regulation of glycolytic flux plays an important role in the acquisition of pluripotency, and more specifically, maintenance and transition between the naïve and primed states.

### Mitochondrial Metabolism

Mitochondria are complex, highly dynamic organelles that are critical for the maintenance of cellular homeostasis, in part due to their canonical role as the major energy generator of the cell. Beyond their role in ATP generation, mitochondria play many other roles within the cell including ROS production, calcium homeostasis, cellular signaling pathways, and synthesis of metabolites, such as fatty acids, amino acids, iron/sulfur clusters, pyrimidines, heme, and steroid hormones (Rizzuto et al., 1993; Dimmer and Scorrano, 2006; de Brito and Scorrano, 2010; Seo et al., 2018). Over the past decade, it has become increasingly recognized that mitochondrial dynamics also significantly impact stem cell function and fate (Liesa and Shirihai, 2013; Labbe et al., 2014; Ma et al., 2015a; Buck et al., 2016; Folmes and Terzic, 2016; Folmes et al., 2016; Khacho et al., 2016; Matilainen et al., 2017). In general, compared to their differentiated counterparts, PSCs have fewer small and globular mitochondria containing poorly developed and immature cristae, that are localized in the perinuclear region (St John et al., 2005; Cho et al., 2006; Lonergan et al., 2006, 2007; Facucho-Oliveira and St John, 2009; Armstrong et al., 2010; Suhr et al., 2010; Folmes et al., 2011; Prigione et al., 2014; St John, 2016). However mitochondrial differences exist between the stages of pluripotency, with primed ESCs containing more elongated mitochondria with better defined cristae compared to their naïve counterparts (Zhou et al., 2012; Ware et al., 2014), despite the observation that primed ESCs have low mitochondrial activity, while naïve ESCs display active mitochondria (Zhou et al., 2012; Takashima et al., 2014; Sperber et al., 2015). Although additional work is required to understand this apparent dichotomy between mitochondrial structure and function, it has been well established that mitochondria and metabolism play a critical role in the naïve-to-primed transition (Zhang et al., 2011; Zhou et al., 2012; Ware et al., 2014; Sperber et al., 2015; Chandel et al., 2016). For instance, a recent study demonstrated that loss of mitochondrial carrier homolog 2 (MTCH2), a direct regulator of mitochondrial fusion/elongation, resulted in less elongated and more fragmented mitochondria in mESCs, leading to delayed naïve-to-primed interconversion. This study indicated that MTCH2 is important for elongated mitochondria which itself was sufficient for driving naïve-to-primed interconversion by altering histone deacetylation and nuclear gene reprogramming (Bahat et al., 2018). In addition, recent work has demonstrated that supplementation with a recombinant truncated human NME7 (NME7_*AB*_) is sufficient to induce a stable naïve-like state in hPSCs (Carter et al., 2016) associated with reactivation of mitochondrial function and stimulation of ATP production (O’Reilly et al., 2019).

Despite the apparently immature mitochondrial structure in PSCs, they maintain high mitochondrial membrane potential, which helps to define their pluripotency and self-renewal characteristics (Schieke et al., 2008; Armstrong et al., 2010; Mah et al., 2011; Prigione et al., 2011). Indeed, mESCs with high mitochondrial membrane potential have the capacity to differentiate into the three germ layers, while those with low mitochondrial membrane potential differentiated mainly into mesodermal cells (Schieke et al., 2008). Furthermore, fully reprogrammed iPSCs appear to have high mitochondrial membrane potential (Folmes et al., 2011) and PSCs may actively maintain this potential by consuming ATP to enable reverse mode ATP synthase activity. Even though the underlying mechanism for maintaining high mitochondrial potential remains to be elucidated, it can impact stem cell function by: (a) maintaining a network of fragmented mitochondria (Mattenberger et al., 2003; Zhang et al., 2011; Teslaa and Teitell, 2015), (b) maintaining redox potential (Shyh-Chang et al., 2011), and (c) priming the cell to provide energy for differentiation (Folmes et al., 2012). Thus, collectively these findings indicate that mitochondria play functional and developmental roles in metabolism of PSCs.

#### Tricarboxylic Acid Cycle

The mitochondrial TCA cycle represents a central hub of energy metabolism, where many pathways involved in central carbon metabolism intersect. Canonically, this cycle predominantly oxidizes its major substrate, pyruvate, to CO_2_ in order to generate reducing factors and electron donors (NADH and FADH_2_) to supply the electron transport chain (ETC) and ATP synthesis. Complementing its role in energy generation, the TCA cycle also maintains a balance between anapleurosis, the reactions to replenish TCA intermediates using predominantly pyruvate and glutamine as substrates, and cataplerosis, whereby partially oxidized intermediates can be extracted to serve as building blocks for anabolic processes including lipid, amino acid and nucleotide biosynthesis, as well as post-translational modification of proteins (Wellen et al., 2009; Boroughs and DeBerardinis, 2015). Through balancing energy generation with cataplerosis to supply substrates for anabolism and post-translational protein modification, the TCA cycle is critical for the regulation of stem cell function and fate.

Pluripotent stem cells appear to have reduced reliance on the canonical role of mitochondria for energy generation, which suggests that they repurpose their mitochondria for other purposes in support of stem cell maintenance. It appears that mitochondrial metabolism in PSCs involves incomplete oxidation of pyruvate, resulting in TCA cycle intermediates that are exported for myriad functions including anabolic reactions and histone modification. In fact, ESCs incompletely oxidize pyruvate through the TCA cycle generating citrate which is then transported and converted to acetyl-CoA through ATP-citrate lyase in the cytoplasm (Wellen et al., 2009; Moussaieff et al., 2015). While fatty acid oxidation (FAO) is known to contribute to the production of mitochondrial acetyl-CoA, its contribution to histone acetylation is limited. Cytosolic acetyl-CoA can serve as a substrate for a number of processes, including acetyl transferases that support the acetylation of many protein classes (Choudhary et al., 2009), as well as a precursor for *de novo* fatty acid synthesis (Wellen and Thompson, 2012; Carey et al., 2015; Moussaieff et al., 2015). It was previously shown that early differentiation of ESCs was correlated to a reduced level of acetyl-CoA production and loss of histone H3 lysine 9 and lysine 27 acetylation, indicating that TCA-derived acetyl-CoA maintains an open chromatin state during pluripotency (Moussaieff et al., 2015). Similarly, it was reported that inhibition of acetyl-CoA resulted in diminished histone acetylation, which in turn stimulated myogenic differentiation (Bracha et al., 2010).

Like other highly proliferative cells, PSCs are dependent on glutamine, which enters the TCA cycle through initial conversion to glutamate by glutaminase, followed by conversion to αKG via glutamate dehydrogenase. αKG is a crucial cofactor for αKG-dependent dioxygenase enzymes, which include JHDMs and TET enzymes (Kaelin and McKnight, 2013). The significance of αKG in regulation of pluripotency through epigenetic modifications has been previously demonstrated by several studies. Addition of cell-permeable dimethyl-αKG (dm-αKG) to culture media was shown to enhance self-renewal, while inhibiting the differentiation of mESCs by promoting histone and DNA demethylation (Carey et al., 2015). The beneficial effect of dm-αKG on pluripotency can be blocked through combined knockdown of the H3K9me2 demethylases, resulting in reduced colony formation. In corroboration with this finding, recent work revealed intracellular αKG can sustain mESCs and hESCs in a glutamine-independent manner (Vardhana et al., 2019). Expression of the pluripotent transcription factors, NANOG or KLF4, in the presence of 2i, resulted in an increased fraction of the TCA cycle intermediates generated from glucose-derived carbon in mESCs and hESCs (Vardhana et al., 2019). Since transient-glutamine depletion eliminated non-pluripotent cells, the authors emphasized that such metabolic rewiring can serve as a selection pressure for pluripotent cells over non-pluripotent cells. A recent study explored a potential functional link between oxidative metabolism, TCA cycle, and αKG on naïve to primed states of mESCs through single-cell analysis (Tischler et al., 2019). The authors demonstrated that isocitrate dehydrogenase 2-mediated production of αKG was critical for sustaining naïve pluripotency in mESCs, even in the absence of 2i. Interestingly, the same study indicates that αKG also contributes to primordial germ cell differentiation (Tischler et al., 2019). This observation is consistent with a previous report that high αKG and αKG-to-succinate ratio promotes differentiation of primed hESCs and mEpiSCs (TeSlaa et al., 2016) through the regulation of histone methylation. This apparent dichotomy between the role of αKG in promoting pluripotency in naïve cells and promoting differentiation in the primed state indicates that metabolite signaling is very dependent on the developmental stage and specific cell type. Overall, the TCA cycle is at the metabolic crossroad contributing to cell fate by coupling its metabolites with both energy production and the chromatin landscape.

#### Oxidative Phosphorylation

Oxidative phosphorylation is a critical pathway for maintaining bioenergetic homeostasis as it links multiple metabolic pathways including glycolysis, the TCA cycle, and FAO with ATP synthesis. This pathway enables electrons donated from NADH/FADH_2_ to flow down reduction potential gradients in the ETC and harnesses the energy produced to pump protons across the inner mitochondrial membrane to develop an electrochemical gradient. The transport of protons back across the membrane and into the mitochondrial matrix is performed by ATP synthase, which couples proton transport with ATP synthesis. Compared to glycolysis, OxPhos is a far more efficient pathway for ATP production, producing 36 ATP molecules per glucose, compared to 2 ATP molecules from glycolysis.

Pluripotent stem cells are typically considered to have a lower rate of OxPhos compared to their differentiated counterparts; however, the relative rate of OxPhos is highly dependent on the specific stage of pluripotency. Naïve PSCs display bivalent metabolism, consisting of both glycolysis and OxPhos, in contrast to primed PSCs which have very low rates of oxygen consumption and are almost entirely dependent on glycolysis despite displaying a more mature mitochondrial phenotype (Zhou et al., 2012; Takashima et al., 2014). Although RNA sequencing and microarrays have shown that mitochondrial electron transport genes are down regulated significantly in primed cells, they retain constant levels of mitochondrial DNA and display more elongated mitochondria with better defined cristae compared to their naïve counterparts (Sperber et al., 2015). Indeed, recent work in canine ESCs (cESCs) has demonstrated that inhibition of complex I of the ETC did not alter the proliferation or viability of primed cESCs, while naïve cESCs were sensitive to complex I inhibition, thus supporting the differences in OxPhos dependency of these pluripotent stages. These metabolic preferences may not only be due to cellular ATP and anabolic demand but may also reflect the availability of oxygen and metabolic substrates during these specific stages *in vivo*. Changes in oxygen consumption have been reported during the transition from mouse preimplantation to early post-implantation development, and while these oxygen tensions are typically not maintained during *in vitro* culture, they may be related to the metabolic preferences of naïve vs. primed PSCs. Indeed, the bivalent metabolic phenotype of naïve PSCs may reflect the metabolic preference of the morula and blastocyst, which utilizes a combination of pyruvate oxidation and glycolysis to meet their metabolic demands, while primed PSCs become almost exclusively dependent on glycolysis, reflecting implantation into the hypoxic uterine wall. Indeed, the importance of reduced oxygen tension has been examined across a number of stem cell populations (Mohyeldin et al., 2010), including a role in improving the acquisition (Yoshida et al., 2009) and maintenance of pluripotency (Ezashi et al., 2005; Forsyth et al., 2006; Prasad et al., 2009; Lengner et al., 2010; Mathieu et al., 2013; Christensen et al., 2015). In part these beneficial effects of physiologically relevant oxygen levels (2–5%) may be due to a reduction in mitochondrial function and oxygen utilization associated with elevated utilization of glucose via glycolysis and amino acid turnover (Forristal et al., 2013; Christensen et al., 2014; Turner et al., 2014; Lees et al., 2015, 2019; Harvey et al., 2016b), although these metabolic changes can occur in the absence of changes in self-renewal (Harvey et al., 2016b). These effects of oxygen may be cell line dependent and may not occur in all iPSC lines, suggesting that metabolic fidelity may represent a marker for PSC and nuclear reprogramming quality (Lees et al., 2015; Harvey et al., 2018; Spyrou et al., 2019). However, the effect of reduced oxygen tension on the transition between naïve and primed pluripotency, as well as the distinct metabolic phenotypes of these states have not been investigated.

Beyond its role in energy generation, the mitochondrial ETC also impacts a number of cellular processes including ROS production (Boveris et al., 1972; Dey et al., 2008), mitochondrial membrane potential (Chen et al., 2014), and mitochondrial protein import (Geissler et al., 2000), which may collectively impact self-renewal and proliferation. While several studies have demonstrated that inhibition of complex I or complex III is associated with impaired cell proliferation (Howell and Sager, 1979; Han et al., 2008; Fendt et al., 2013; Wheaton et al., 2014), the mechanism by which the ETC regulates cell proliferation has only recently been elucidated. Using a CRISPR-based genetic screen Birsoy et al. (2015) revealed that the ETC enables the synthesis of aspartate, which is a precursor for purine and pyrimidine syntheses. To functionally validate the genetic screen, they demonstrated that supplementation with exogenous aspartate or overexpression of aspartate transporter enabled cells without ETC activity to proliferate, while loss of cytosolic aspartate aminotransferase (GOT1), which consumes aspartate to transfer electrons into mitochondria, resulted in cell death upon ETC inhibition. Collectively, this indicates that in addition to the canonical role of mitochondrial ETC in ATP synthesis, it also supports cell proliferation through the generation of aspartate, which functions as an anabolic substrate. In summary, both naïve and primed PSCs have active OxPhos, but the extent to which they rely on OxPhos for energy production and proliferation appears to differ due to the variances in the availability of oxygen and other metabolites that can sustain the high energetic demand of proliferation in PSCs.

### Lipid Metabolism

Lipids play vital roles in the maintenance of cellular homeostasis by serving as energy sources, signaling entities and building blocks for membranes. Lipid metabolism represents a carefully regulated balance between catabolism (FAO) and anabolism (*de novo* biosynthesis), which is highly dependent on the metabolic requirements of a specific cell state. FAO consists of active transport of medium and long chain fatty acids into the mitochondria through a regulated carnitine palmitoyl transferase system, with subsequent oxidation by the chain-length specific enzymes of beta-oxidation to generate acetyl-CoA that feeds into the TCA cycle and NADH that donates its electrons to the ETC. In contrast, *de novo* fatty acid biosynthesis requires substrates from multiple metabolic pathways, including acetyl-CoA, reducing factors and ATP, in order to build essential fatty acids. Despite the essential role of lipids in cellular homeostasis, the impact of lipids on PSC maintenance and self-renewal remains relatively unexplored in comparison with other metabolic pathways. Recent studies have begun to examine the impact of lipid availability on hPSCs metabolism and function (Zhang et al., 2016a; Cornacchia et al., 2019) and demonstrated that lipid replete media (E8 and to a lesser extend mTeSR) significantly remodeled the metabolic state in order to sustain lipogenesis (Zhang et al., 2016a). The metabolic reprogramming induced by lipid deficiency significantly increased the oxidative PPP to support NADPH regeneration, increased glutamine consumption and fatty acid biosynthesis, at the expense of oxidative metabolism, which is consistent with the importance of glutaminolysis and the PPP to lipid biosynthesis in PSCs (Varum et al., 2011; Tohyama et al., 2016).

Lipid availability has also recently been shown to regulate the transition between naïve and primed hPSCs, with E8 medium inducing a primed-to-naïve intermediate state of pluripotency associated with increased *de novo* lipogenesis (Cornacchia et al., 2019). The intermediate state in E8-hPSCs recapitulated many of the features of naïve pluripotency, however there were key differences, including a moderate state of DNA hypomethylation, specifically in terms of a global reduction in H3K27me3 and H3K9me3 levels, a known feature of naïve human PSCs. This intermediate state is dependent on lipid-free culture conditions, which promotes active lipid biosynthesis and endogenous ERK inhibition, features which are lost upon lipid supplementation. Interestingly, transcriptional analysis of E8-hPSCs and pre-implantation epiblasts demonstrated that *de novo* lipogenesis is a consistent transcriptional feature across *in vivo* and *in vitro* naïve pluripotency. The lipogenic state also supports the metabolic remodeling of the epigenome, including hyperacetylation of H3K27, H3K9 and H4K8 associated with increased acetyl-CoA metabolism and hypomethylation of DNA due to an increase in αKG to succinate ratio that activates JMJD and TET chromatin demethylases and a reduction in the SAM to S-adenosylhomocysteine (SAH) ratio. Indeed, this metabolic and epigenetic remodeling also increased the propensity for neuroectodermal differentiation, which is consistent with the observation that fatty acid synthase dependent-*de novo* lipogenesis is essential for neural stem cell proliferation and neurogenesis (Knobloch et al., 2013). Therefore, this data supports the concept that baseline pluripotent culture conditions have downstream effects on differentiation capacity.

In addition to their metabolic roles, lipids may also regulate the primed-to-naïve conversion as a signaling molecule (Kime et al., 2016). Lysophosphatidic acid (LPA) lipid signaling and the LPA-producing enzyme autotoxin have been implicated in establishing naïve PSCs in coordination with LIF and bone morphogenetic protein 4 (BMP4) signaling (Kime et al., 2016). These elegant studies support the importance of nutrient availability for defining stem cell features and suggest that manipulating energy metabolism may be sufficient to promote the transition between stem cell states. These studies indicate that metabolism is intertwined with epigenetics and the transcriptional landscape of PSCs, and additional work is required to determine how these pathways ultimately define the fate of PSCs.

### Amino Acid Metabolism

Amino acids are important substrates for the biosynthesis of the basic building blocks of the cell, including proteins, lipids and nucleotides (Locasale, 2013), and as such have been demonstrated to contribute significantly to the maintenance of pluripotency and stem cell fate. Beyond the direct anabolic roles of amino acids, such as *de novo* purine biosynthesis, amino acid metabolism is intimately related to one-carbon metabolism, which consists of the methionine and folate cycles that maintain cellular pools of one-carbon residues associated with S-adenosylmethionine (SAM) and folate (Clare et al., 2019). This one-carbon pool is not only essential for donating methyl groups for the synthesis of amino acids, nucleotides and phospholipids, but SAM also represents the principal substrate for post-translational methylation of RNA, DNA, and proteins, making it a critical connection between changes in metabolism and remodeling of the epigenetic state of the cell.

mESCs are highly dependent on threonine metabolism to maintain their pluripotency and self-renewal (Wang et al., 2009). Threonine dehydrogenase (TDH) and downstream enzymes in threonine metabolism, glycine c-acetyltransferase (GCAT) and glycine decarboxylase (GLDC), are highly expressed in mESCs and are rapidly downregulated during differentiation. Indeed, inhibition of TDH or complete removal of threonine from cell culture medium results in loss of stemness, reduced proliferation, apoptosis, and cell cycle arrest (Wang et al., 2009; Alexander et al., 2011), while L-threonine supplementation and induction of TDH supports induction of pluripotency through nuclear reprogramming (Ryu and Han, 2011; Han et al., 2013; Chen and Wang, 2014). The downstream effects of threonine metabolism on stem cell fate can be contributed to a number of mechanisms, including supplying methyl groups to the one carbon metabolism pool for the biosynthesis of cellular building blocks, as well as maintaining a high ratio of SAM to SAH to promote H3K4me3, which is critical for the maintenance of the pluripotent state (Bernstein et al., 2006; Wang et al., 2009, 2011; Shyh-Chang et al., 2013b). L-threonine may mediate some of its proliferative effects through lipid raft/caveolae-dependent regulation of ERK, p38, JNK/SAPK, and mTORC pathways (Ryu and Han, 2011). In humans, TDH is only expressed as a non-functional pseudogene, thus hESCs rely on methionine metabolism in the same way that mESCs rely on threonine to maintain a high ratio of SAM/SAH (Shiraki et al., 2014). Interestingly, a recent study has also implicated methionine in mESC maintenance downstream of SIRT1 expression (Tang et al., 2017). SIRT1 KO mESCs display an elevated ratio of methionine/SAM due to a reduction in the expression of methionine adenosyltransferase 2a (MAT2a), which catalyzes the conversion of methionine to SAM (Tang et al., 2017). Regulation of methionine metabolism appears to be in part through a SIRT1-dependent protein expression of c-MYC and n-MYC, which bind to the MAT2a promoter and induce its expression. H3k27me3 is also regulated in the naïve state by N-methyltransferase, which consumes SAM, making it unavailable for histone methylation (Sperber et al., 2015).

Proline may also play an important role in regulating PSC identity, as a feedback loop has been identified whereby proline modulates the GCN2-EIF2a-ATF4 amino acid starvation response pathway, which in turn suppresses proline biosynthesis to restrict proliferation and maintain ESC identity (D’Aniello et al., 2015). Supplementation of mESC culture media with proline is sufficient to promote cell proliferation and transition of mESCs to EpiSCs (Washington et al., 2010; Casalino et al., 2011), and induce ESCs into a mesenchymal-like, motile phenotype (Comes et al., 2013; D’Aniello et al., 2015). Interestingly, this effect is completely reversible, as removal of proline results in the restoration of the mESC state. These phenotypic changes may in part be due to an increase in global H3K9 and H3K36 methylation, and can be reversed using vitamin C which promotes the demethylation of these marks (Comes et al., 2013). Ornithine supplementation can also induce a mESC to EpiSC transition, suggesting that this phenotype may be mediated through a common intermediate in the catabolism of proline and ornithine, namely Δ^1^-pyrroline-5-carboxylate (Casalino et al., 2011). Collectively, these studies highlight the importance of proline in ESC identity, but further studies are required to decipher the underlying mechanism by which this amino acid impacts stem cell identity.

## Energy Metabolism Drives Acquisition of Pluripotency Through Nuclear Reprogramming

**FIGURE 4:**
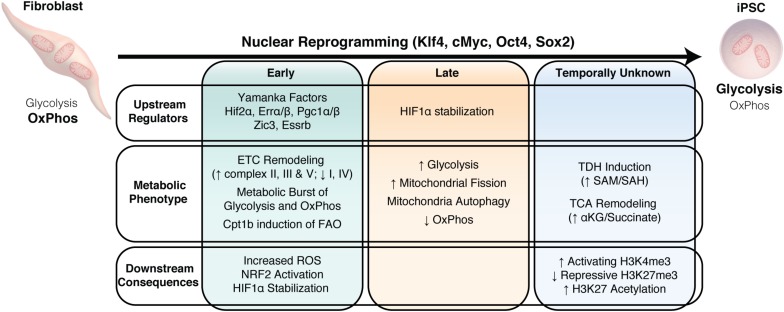
Metabolic remodeling drives nuclear reprogramming. Summary of the temporal changes in upstream regulators, the downstream metabolic changes and the consequences of this metabolic phenotype on nuclear reprogramming.

Multiple studies have now demonstrated that remodeling of energy metabolism plays a critical role early during nuclear reprogramming of somatic cells into iPSCs (Takahashi and Yamanaka, 2006), indicating that metabolic reprogramming in not simply a consequence on cell transition, but a driving force (Figure 4). Upregulation of glycolysis has been demonstrated to precede the induction of pluripotency markers and is a critical component for successful nuclear reprogramming (Folmes et al., 2011). Indeed cells that have a glycolytic phenotype reprogram more efficiently than those with greater reliance on OxPhos (Panopoulos et al., 2012) and stimulation of glycolysis enhances reprogramming, while inhibition of glycolytic or stimulation of OxPhos suppresses reprogramming (Yoshida et al., 2009; Esteban et al., 2010; Zhu et al., 2010; Folmes et al., 2011; Prigione et al., 2014). Changes in protein expression of ETC subunits are among the earliest changes that occur during nuclear reprogramming (Hansson et al., 2012), consisting of decreased expression of complex I and IV subunits and increased expression of complex II, III and V subunits. This reorganization of the ETC, particularly the reduced expression of complex I and increased expression of complex II, suggests that FADH_2_ may be the primary electron donor for ETC function during nuclear reprogramming and in the resultant iPSCs. Functionally, this manifests as a transient burst of OxPhos activity that accompanies the acceleration of glycolysis at the early stage of reprogramming (Prigione et al., 2014; Kida et al., 2015; Hawkins et al., 2016). Indeed, increased ROS generated from OxPhos during this period may be critical for driving the metabolic transition during nuclear reprogramming through modification of cysteine residues on the NRG2 repressor protein KEAP1, leading to NRF2 activation, which subsequently activates HIF1α to support glycolysis (Mathieu et al., 2014; Hawkins et al., 2016). In addition, ZIC3 and ESSRB may act synergistically to promote glycolysis in a HIF-independent mechanism, while offsetting their opposing effects on OxPhos to promote both reprogramming and the conversion of primed PSCs into the naïve state (Sone et al., 2017). As reprogramming progresses, the abundance of mitochondrial DNA and mass gradually decrease as cells undergo autophagic mitochondrial clearance resulting in fewer spherical mitochondria with poorly developed and immature cristae compared to their parental fibroblasts (Folmes et al., 2011; Prigione et al., 2011; Ma et al., 2015b). Mitochondrial fusion/fission dynamics are crucial for somatic cell nuclear reprogramming. Inhibition of the mitochondrial fission protein DRP1 is sufficient to suppress the early stage of reprogramming of somatic cells (Vazquez-Martin et al., 2012), while expression of mitochondrial fusion proteins, such as MFN1 and MFN2, appears to be a barrier for reprogramming (Son et al., 2015). Inhibition of MFN1/MFN2 increases reprogramming efficiency by activating RAS/RAF signaling to enable ROS-mediated HIF1α stabilization to facilitate the transition of OxPhos to glycolytic metabolism (Son et al., 2015). In summary, efficient nuclear reprogramming involves complex and stage specific metabolic changes driven by OxPhos and glycolysis.

Although metabolic remodeling has broadly focused on the transition from predominantly OxPhos to higher glycolytic activity upon nuclear reprogramming (Folmes et al., 2011, 2013a; Prigione et al., 2014; Cao et al., 2015), alternative metabolic pathways, including amino acid and lipid metabolism, are increasingly being identified to play an important role during nuclear reprogramming. As discussed earlier, threonine and methionine are indispensable amino acids to maintain the one-carbon pool that is essential for donating methyl groups for both anabolism and post-translation modifications. Indeed, L-threonine supplementation and induction of TDH supports induction of pluripotency through nuclear reprogramming by promoting H3K4me3 (Ryu and Han, 2011; Han et al., 2013; Chen and Wang, 2014). Recent evidence has also implicated GLDC, a key enzyme in the glycine cleavage, in early metabolic remodeling during nuclear reprogramming and maintenance of the pluripotent state (Kang et al., 2019). GLDC expression occurs early in the reprogramming process and appears to be downstream of the reprogramming factors KLF4 and c-MYC, and reprogramming efficiency can be impaired with GLDC knockdown and enhanced with GLDC overexpression. GLDC knockdown in mESCs leads to a reduction in a number of glycolytic intermediates and the loss of self-renewal, suggesting that GLDC functions in reprogramming by regulating glycolysis (Kang et al., 2019).

*De novo* fatty acid synthesis also appears critical for acquisition and maintenance of pluripotency. Nuclear reprogramming is accompanied by enhanced lipogenesis due to increased expression of fatty acid synthase (FAS) and acetyl-CoA carboxylase 1 (ACC1) (Wang et al., 2017), and supplementation with exogenous oleic acid can increase reprogramming efficiency. Mechanistically, increased lipogenesis via ACC1 leads to enhanced mitochondrial fission by competing for a limited acetyl-CoA pool, thus blocking acetylation-dependent degradation of the mitochondrial fission 1 protein (FIS1) via the ubiquitin-proteasome pathway. In addition, increased lipid generation also drives mitochondrial dynamics toward a fission phenotype, which ultimately promotes nuclear reprogramming. Interestingly, FAO also appears to regulate nuclear reprogramming, consistent with its crucial role for oocyte and early embryo development (Oey et al., 2005; Dunning et al., 2010; Shyh-Chang et al., 2013a). Long-chain FAO is primarily regulated by the transport of long-chain fatty acids (LCFA) across the inner mitochondrial membrane by the carnitine palmitoyltransferase (CPT) system, containing CPT1 and CPT2. CPT1b, which catalyzes the rate limiting transfer of the acyl group from coenzyme A to carnitine, was shown to be significantly upregulated at the early stage of reprogramming and overexpression of CPT1b improved reprogramming efficiency (Lin et al., 2018). Addition of palmitoylcarnitine (PC), the product of CPT1, was sufficient to stimulate OxPhos activity and enhance reprogramming only during the first 3 days of reprogramming, after which OxPhos was suppressed with PC supplementation, suggesting that the importance of FAO to reprogram may coincide with the hypermetabolic state observed early in the reprogramming process.

Although significant advances have been made in nuclear reprogramming technology, incomplete reprogramming can generate iPSCs that have transcriptional, epigenetic, and metabolic memory of their parental source (Chan et al., 2009; Kim et al., 2010; Ohi et al., 2011; Lee et al., 2013; Spyrou et al., 2019). While the glycolytic phenotype of iPSCs and ESCs are in general very similar, differences have been identified in specific metabolites in human (polyunsaturated fatty acids, SAM) and mouse (phosphatidylcholine, phosphatidylethanolamine, amino acids and metabolites in polyamine biosynthesis) iPSCs compared to ESCs (Meissen et al., 2012; Panopoulos et al., 2012). Although these differences exist, their functional impact on maintenance of pluripotency or differentiation capacity have not been examined. Partially reprogrammed cells, which are characterized by forming stable ESC-like colonies but not expressing endogenous OCT4, NANOG, SSEA4 and TRA-1-60, appear to have a distinct mitochondria and metabolic profile that are intermediate between fully reprogrammed iPSCs/ESCs and parental fibroblasts in terms of mitochondrial morphology and gene expression, and concentrations of glycolytic and OxPhos intermediates (Lee et al., 2016; Park et al., 2017). This insufficient repression of mitochondrial function and activation of glycolysis in partially reprogrammed cells can be rescued through microRNA 31 overexpression, which suppresses succinate dehydrogenase A activity to promote the transition from OxPhos to glycolysis and enhance reprogramming efficiency (Lee et al., 2016). Interestingly, recent evidence also indicates that dysregulated mitochondria fusion/fission dynamics impairs the ability to achieve full pluripotency and restricts developmental potential of iPSCs (Zhong et al., 2019). Therefore, strategies that optimize mitochondrial and metabolic remodeling during nuclear reprogramming may improve reprogramming fidelity and ultimately their use in downstream applications.

Collectively, these studies demonstrate that complex and coordinated remodeling of energy metabolism is critical to drive efficient nuclear reprogramming, in part thought the resetting of the epigenetic landscape. While this appears to be the case in nuclear reprogramming, early metabolic remodeling may also play an important role in other cell state transitions, including from naive to primed pluripotency and release from pluripotency along lineage specific differentiation.

## Summary

Over the past decade, significant advances have been made in our understanding of the metabolic requirements of stem cells and the important role that energy metabolism plays in regulating stem cell function and fate. An emerging concept supports metabolism as not simply a homeostatic system that matches energy supply with energetic demands, but a critical early step during transition between cellular states. This concept is backed by two observations: a) metabolic changes are often some of the earliest to occur during cell fate transitions and b) there is growing appreciation that metabolic pathways directly contribute to the epigenetic remodeling of the cell. This realization has placed emphasis on understanding the microenvironment in which these cells are cultured, as numerous studies have now shown that modifying availability of a single metabolic substrate can dramatically impact stem cell identity. As the field increasingly dissects the finer metabolic distinctions between pluripotent states and discovers how these interact with regulators of stem cell fate, it will be interesting to see if manipulation of substrate supply and energy metabolism may help to enable the derivation and culture of other PSC states, such as totipotent stem cells or formative PSCs. In addition, modulation of energy metabolism may ultimately be harnessed for translational and clinical applications, not only to support manufacturing and lineage specification of PSCs for regenerative therapies, but also to promote innate regenerative capacity and augment current cell-based therapies.

## Author Contributions

All authors listed have made a substantial, direct and intellectual contribution to the work, and approved it for publication.

## Conflict of Interest

The authors declare that the research was conducted in the absence of any commercial or financial relationships that could be construed as a potential conflict of interest.

## References

[B1] Alexander P. B. Wang J. McKnight S. L. (2011). Targeted killing of a mammalian cell based upon its specialized metabolic state. *Proc. Natl. Acad. Sci. U.S.A.* 108 15828–15833. 10.1073/pnas.1111312108 21896756 PMC3179072

[B2] Altshuler A. Verbuk M. Bhattacharya S. Abramovich I. Haklai R. Hanna J. H. (2018). RAS regulates the transition from naive to primed pluripotent stem cells. *Stem Cell Rep.* 10 1088–1101. 10.1016/j.stemcr.2018.01.004 29456180 PMC5918191

[B3] Armstrong L. Tilgner K. Saretzki G. Atkinson S. P. Stojkovic M. Moreno R. (2010). Human induced pluripotent stem cell lines show similar stress defence mechanisms and mitochondrial regulation to human embryonic stem cells. *Stem Cells* 28 661–673. 10.1002/stem.307 20073085

[B4] Bahat A. Goldman A. Zaltsman Y. Khan D. H. Halperin C. Amzallag E. (2018). MTCH2-mediated mitochondrial fusion drives exit from naive pluripotency in embryonic stem cells. *Nat. Commun.* 9:5132. 10.1038/s41467-018-07519-w 30510213 PMC6277412

[B5] Barbehenn E. K. Wales R. G. Lowry O. H. (1978). Measurement of metabolites in single preimplantation embryos; a new means to study metabolic control in early embryos. *J. Embryol. Exp. Morphol.* 43 29–46. 580293

[B6] Bernstein B. E. Mikkelsen T. S. Xie X. Kamal M. Huebert D. J. Cuff J. (2006). A bivalent chromatin structure marks key developmental genes in embryonic stem cells. *Cell* 125 315–326. 10.1016/j.cell.2006.02.041 16630819

[B7] Birsoy K. Wang T. Chen W. W. Freinkman E. Abu-Remaileh M. Sabatini D. M. (2015). An essential role of the mitochondrial electron transport chain in cell proliferation is to enable aspartate synthesis. *Cell* 162 540–551. 10.1016/j.cell.2015.07.016 26232224 PMC4522279

[B8] Boroughs L. K. DeBerardinis R. J. (2015). Metabolic pathways promoting cancer cell survival and growth. *Nat. Cell Biol.* 17 351–359. 10.1038/ncb3124 25774832 PMC4939711

[B9] Boveris A. Oshino N. Chance B. (1972). The cellular production of hydrogen peroxide. *Biochem. J.* 128 617–630. 10.1042/bj1280617 4404507 PMC1173814

[B10] Bracha A. L. Ramanathan A. Huang S. Ingber D. E. Schreiber S. L. (2010). Carbon metabolism-mediated myogenic differentiation. *Nat. Chem. Biol.* 6 202–204. 10.1038/nchembio.301 20081855 PMC2822028

[B11] Brons I. G. Smithers L. E. Trotter M. W. Rugg-Gunn P. Sun B. Chuva de Sousa Lopes S. M. (2007). Derivation of pluripotent epiblast stem cells from mammalian embryos. *Nature* 448 191–195. 10.1038/nature05950 17597762

[B12] Buck M. D. O’Sullivan D. Klein Geltink R. I. Curtis J. D. Chang C. H. Sanin D. E. (2016). Mitochondrial dynamics controls t cell fate through metabolic programming. *Cell* 166 63–76. 10.1016/j.cell.2016.05.035 27293185 PMC4974356

[B13] Cao Y. Guo W. T. Tian S. He X. Wang X. W. Liu X. (2015). miR-290/371-Mbd2-Myc circuit regulates glycolytic metabolism to promote pluripotency. *EMBO J.* 34 609–623. 10.15252/embj.201490441 25603933 PMC4365031

[B14] Carey B. W. Finley L. W. Cross J. R. Allis C. D. Thompson C. B. (2015). Intracellular alpha-ketoglutarate maintains the pluripotency of embryonic stem cells. *Nature* 518 413–416. 10.1038/nature13981 25487152 PMC4336218

[B15] Carter M. G. Smagghe B. J. Stewart A. K. Rapley J. A. Lynch E. Bernier K. J. (2016). A Primitive growth factor, NME7AB, Is sufficient to induce stable naive state human pluripotency; reprogramming in this novel growth factor confers superior differentiation. *Stem Cells* 34 847–859. 10.1002/stem.2261 26749426

[B16] Casalino L. Comes S. Lambazzi G. De Stefano B. Filosa S. De Falco S. (2011). Control of embryonic stem cell metastability by L-proline catabolism. *J. Mol. Cell Biol.* 3 108–122. 10.1093/jmcb/mjr001 21307025

[B17] Cha Y. Han M.-J. Cha H.-J. Zoldan J. Burkart A. Jung J. H. (2017). Metabolic control of primed human pluripotent stem cell fate and function by the miR-200c–SIRT2 axis. *Nat. Cell Biol.* 19 445–456. 10.1038/ncb3517 28436968 PMC5545746

[B18] Chan E. M. Ratanasirintrawoot S. Park I. H. Manos P. D. Loh Y. H. Huo H. (2009). Live cell imaging distinguishes bona fide human iPS cells from partially reprogrammed cells. *Nat. Biotechnol.* 27 1033–1037. 10.1038/nbt.1580 19826408

[B19] Chandel N. S. Jasper H. Ho T. T. Passegue E. (2016). Metabolic regulation of stem cell function in tissue homeostasis and organismal ageing. *Nat. Cell Biol.* 18 823–832. 10.1038/ncb3385 27428307

[B20] Chen G. Wang J. (2014). Threonine metabolism and embryonic stem cell self-renewal. *Curr. Opin. Clin. Nutr. Metab. Care* 17 80–85. 10.1097/MCO.0000000000000007 24232288

[B21] Chen W. W. Birsoy K. Mihaylova M. M. Snitkin H. Stasinski I. Yucel B. (2014). Inhibition of ATPIF1 ameliorates severe mitochondrial respiratory chain dysfunction in mammalian cells. *Cell Rep.* 7 27–34. 10.1016/j.celrep.2014.02.046 24685140 PMC4040975

[B22] Cho Y. M. Kwon S. Pak Y. K. Seol H. W. Choi Y. M. Park do J. (2006). Dynamic changes in mitochondrial biogenesis and antioxidant enzymes during the spontaneous differentiation of human embryonic stem cells. *Biochem. Biophys. Res. Commun.* 348 1472–1478. 10.1016/j.bbrc.2006.08.020 16920071

[B23] Choi Y. J. Lin C. P. Risso D. Chen S. Kim T. A. Tan M. H. (2017). Deficiency of microRNA miR-34a expands cell fate potential in pluripotent stem cells. *Science* 355:6325. 10.1126/science.aag1927 28082412 PMC6138252

[B24] Choudhary C. Kumar C. Gnad F. Nielsen M. L. Rehman M. Walther T. C. (2009). Lysine acetylation targets protein complexes and co-regulates major cellular functions. *Science* 325 834–840. 10.1126/science.1175371 19608861

[B25] Christensen D. R. Calder P. C. Houghton F. D. (2014). Effect of oxygen tension on the amino acid utilisation of human embryonic stem cells. *Cell. Physiol. Biochem.* 33 237–246. 10.1159/000356665 24496287

[B26] Christensen D. R. Calder P. C. Houghton F. D. (2015). GLUT3 and PKM2 regulate OCT4 expression and support the hypoxic culture of human embryonic stem cells. *Sci. Rep.* 5:154. 10.1038/srep17500 26639784 PMC4671001

[B27] Clare C. E. Brassington A. H. Kwong W. Y. Sinclair K. D. (2019). One-carbon metabolism: linking nutritional biochemistry to epigenetic programming of long-term development. *Annu. Rev. Anim. Biosci.* 7 263–287. 10.1146/annurev-animal-020518-115206 30412672

[B28] Comes S. Gagliardi M. Laprano N. Fico A. Cimmino A. Palamidessi A. (2013). L-Proline induces a mesenchymal-like invasive program in embryonic stem cells by remodeling H3K9 and H3K36 methylation. *Stem Cell Rep.* 1 307–321. 10.1016/j.stemcr.2013.09.001 24319666 PMC3849245

[B29] Cornacchia D. Zhang C. Zimmer B. Chung S. Y. Fan Y. Soliman M. A. (2019). Lipid deprivation induces a stable, naive-to-primed intermediate state of pluripotency in human PSCs. *Cell Stem Cell* 12:e110. 10.1016/j.stem.2019.05.001 31155483 PMC7549840

[B30] D’Aniello C. Fico A. Casalino L. Guardiola O. Di Napoli G. Cermola F. (2015). A novel autoregulatory loop between the Gcn2-Atf4 pathway and (L)-Proline metabolism controls stem cell identity. *Cell Death Differ.* 22 1094–1105. 10.1038/cdd.2015.24 25857264 PMC4572871

[B31] Davidson K. C. Mason E. A. Pera M. F. (2015). The pluripotent state in mouse and human. *Development* 142 3090–3099. 10.1242/dev.116061 26395138

[B32] de Brito O. M. Scorrano L. (2010). An intimate liaison: spatial organization of the endoplasmic reticulum-mitochondria relationship. *EMBO J* 29 2715–2723. 10.1038/emboj.2010.177 20717141 PMC2924651

[B33] Dey B. K. Stalker L. Schnerch A. Bhatia M. Taylor-Papidimitriou J. Wynder C. (2008). The histone demethylase KDM5b/JARID1b plays a role in cell fate decisions by blocking terminal differentiation. *Mol. Cell Biol.* 28 5312–5327. 10.1128/MCB.00128-08 18591252 PMC2519745

[B34] Dimmer K. S. Scorrano L. (2006). Deconstructing mitochondria: what for? *Physiology* 21 233–241. 10.1152/physiol.00010.2006 16868312

[B35] Dunning K. R. Cashman K. Russell D. L. Thompson J. G. Norman R. J. Robker R. L. (2010). Beta-oxidation is essential for mouse oocyte developmental competence and early embryo development. *Biol. Reprod.* 83 909–918. 10.1095/biolreprod.110.084145 20686180

[B36] Esteban M. A. Wang T. Qin B. Yang J. Qin D. Cai J. (2010). Vitamin C enhances the generation of mouse and human induced pluripotent stem cells. *Cell Stem Cell* 6 71–79. 10.1016/j.stem.2009.12.001 20036631

[B37] Evans M. J. Kaufman M. H. (1981). Establishment in culture of pluripotential cells from mouse embryos. *Nature* 292 154–156. 10.1038/292154a0 7242681

[B38] Ezashi T. Das P. Roberts R. M. (2005). Low O_2_ tensions and the prevention of differentiation of hES cells. *Proc. Natl. Acad. Sci. U.S.A.* 102 4783–4788. 10.1073/pnas.0501283102 15772165 PMC554750

[B39] Facucho-Oliveira J. M. St John J. C. (2009). The relationship between pluripotency and mitochondrial DNA proliferation during early embryo development and embryonic stem cell differentiation. *Stem Cell Rev. Rep.* 5 140–158. 10.1007/s12015-009-9058-0 19521804

[B40] Fendt S. M. Bell E. L. Keibler M. A. Davidson S. M. Wirth G. J. Fiske B. (2013). Metformin decreases glucose oxidation and increases the dependency of prostate cancer cells on reductive glutamine metabolism. *Cancer Res.* 73 4429–4438. 10.1158/0008-5472.CAN-13-0080 23687346 PMC3930683

[B41] Flores A. Schell J. Krall A. S. Jelinek D. Miranda M. Grigorian M. (2017). Lactate dehydrogenase activity drives hair follicle stem cell activation. *Nat. Cell Biol.* 19 1017–1026. 10.1038/ncb3575 28812580 PMC5657543

[B42] Folmes C. D. Dzeja P. P. Nelson T. J. Terzic A. (2012). Metabolic plasticity in stem cell homeostasis and differentiation. *Cell Stem Cell* 11 596–606. 10.1016/j.stem.2012.10.002 23122287 PMC3593051

[B43] Folmes C. D. Ma H. Mitalipov S. Terzic A. (2016). Mitochondria in pluripotent stem cells: stemness regulators and disease targets. *Curr. Opin. Genet. Dev.* 38 1–7. 10.1016/j.gde.2016.02.001 26953561 PMC5011451

[B44] Folmes C. D. Martinez-Fernandez A. Faustino R. S. Yamada S. Perez-Terzic C. Nelson T. J. (2013a). Nuclear reprogramming with c-Myc potentiates glycolytic capacity of derived induced pluripotent stem cells. *J. Cardiovasc. Transl. Res.* 6 10–21. 10.1007/s12265-012-9431-2 23247633 PMC3750736

[B45] Folmes C. D. Nelson T. J. Martinez-Fernandez A. Arrell D. K. Lindor J. Z. Dzeja P. P. (2011). Somatic oxidative bioenergetics transitions into pluripotency-dependent glycolysis to facilitate nuclear reprogramming. *Cell Metab.* 14 264–271. 10.1016/j.cmet.2011.06.011 21803296 PMC3156138

[B46] Folmes C. D. Park S. Terzic A. (2013b). Lipid metabolism greases the stem cell engine. *Cell Metab.* 17 153–155. 10.1016/j.cmet.2013.01.010 23395162

[B47] Folmes C. D. Terzic A. (2014). Metabolic determinants of embryonic development and stem cell fate. *Reprod. Fertil. Dev.* 27 82–88. 10.1071/rd14383 25472047 PMC4444364

[B48] Folmes C. D. Terzic A. (2016). Energy metabolism in the acquisition and maintenance of stemness. *Semin. Cell Dev. Biol.* 52 68–75. 10.1016/j.semcdb.2016.02.010 26868758 PMC4905551

[B49] Forristal C. E. Christensen D. R. Chinnery F. E. Petruzzelli R. Parry K. L. Sanchez-Elsner T. (2013). Environmental oxygen tension regulates the energy metabolism and self-renewal of human embryonic stem cells. *PLoS One* 8:e62507. 10.1371/journal.pone.0062507 23671606 PMC3645991

[B50] Forsyth N. R. Musio A. Vezzoni P. Simpson A. H. Noble B. S. McWhir J. (2006). Physiologic oxygen enhances human embryonic stem cell clonal recovery and reduces chromosomal abnormalities. *Clon. Stem Cell* 8 16–23. 10.1089/clo.2006.8.16 16571074

[B51] Gafni O. Weinberger L. Mansour A. A. Manor Y. S. Chomsky E. Ben-Yosef D. (2013). Derivation of novel human ground state naive pluripotent stem cells. *Nature* 504 282–286. 10.1038/nature12745 24172903

[B52] Gardner D. K. (1998). Changes in requirements and utilization of nutrients during mammalian preimplantation embryo development and their significance in embryo culture. *Theriogenology* 49 83–102. 10.1016/S0093-691x(97)00404-4 10732123

[B53] Gardner D. K. Harvey A. J. (2015). Blastocyst metabolism. *Reprod. Fertil. Dev.* 27 638–654. 10.1071/rd14421 25751298

[B54] Geissler A. Krimmer T. Bomer U. Guiard B. Rassow J. Pfanner N. (2000). Membrane potential-driven protein import into mitochondria. The sorting sequence of cytochrome b(2) modulates the deltapsi-dependence of translocation of the matrix-targeting sequence. *Mol. Biol. Cell* 11 3977–3991. 10.1091/mbc.11.11.3977 11071921 PMC15051

[B55] Gu W. Gaeta X. Sahakyan A. Chan A. B. Hong C. S. Kim R. (2016). Glycolytic metabolism plays a functional role in regulating human pluripotent stem cell state. *Cell Stem Cell* 19 476–490. 10.1016/j.stem.2016.08.008 27618217 PMC5055460

[B56] Guo G. von Meyenn F. Santos F. Chen Y. Reik W. Bertone P. (2016). Naive pluripotent stem cells derived directly from isolated cells of the human inner cell mass. *Stem Cell Rep.* 6 437–446. 10.1016/j.stemcr.2016.02.005 26947977 PMC4834040

[B57] Guppy M. Greiner E. Brand K. (1993). The role of the Crabtree effect and an endogenous fuel in the energy metabolism of resting and proliferating thymocytes. *Eur. J. Biochem.* 212 95–99. 10.1111/j.1432-1033.1993.tb17637.x 8444168

[B58] Han C. Gu H. Wang J. Lu W. Mei Y. Wu M. (2013). Regulation of L-threonine dehydrogenase in somatic cell reprogramming. *Stem Cell* 31 953–965. 10.1002/stem.1335 23355387

[B59] Han Y. H. Kim S. H. Kim S. Z. Park W. H. (2008). Antimycin A as a mitochondrial electron transport inhibitor prevents the growth of human lung cancer A549 cells. *Oncol. Rep.* 20 689–693. 18695925

[B60] Hansson J. Rafiee M. R. Reiland S. Polo J. M. Gehring J. Okawa S. (2012). Highly coordinated proteome dynamics during reprogramming of somatic cells to pluripotency. *Cell Rep.* 2 1579–1592. 10.1016/j.celrep.2012.10.014 23260666 PMC4438680

[B61] Harvey A. Caretti G. Moresi V. Renzini A. Adamo S. (2019). Interplay between metabolites and the epigenome in regulating embryonic and adult stem cell potency and maintenance. *Stem Cell Rep.* 13 573–589. 10.1016/j.stemcr.2019.09.003 31597110 PMC6830055

[B62] Harvey A. J. O’Brien C. Lambshead J. Sheedy J. R. Rathjen J. Laslett A. L. (2018). Physiological oxygen culture reveals retention of metabolic memory in human induced pluripotent stem cells. *PLoS One* 13:e0193949. 10.1371/journal.pone.0193949 29543848 PMC5854358

[B63] Harvey A. J. Rathjen J. Gardner D. K. (2016a). Metaboloepigenetic regulation of pluripotent stem cells. *Stem Cell Int.* 2016 1–15. 10.1155/2016/1816525 26839556 PMC4709785

[B64] Harvey A. J. Rathjen J. Yu L. J. Gardner D. K. (2016b). Oxygen modulates human embryonic stem cell metabolism in the absence of changes in self-renewal. *Reprod. Fertil. Dev.* 28 446–458. 10.1071/rd14013 25145274

[B65] Hawkins K. E. Joy S. Delhove J. M. Kotiadis V. N. Fernandez E. Fitzpatrick L. M. (2016). NRF2 orchestrates the metabolic shift during induced pluripotent stem cell reprogramming. *Cell Rep.* 14 1883–1891. 10.1016/j.celrep.2016.02.003 26904936 PMC4785773

[B66] Howell N. Sager R. (1979). Cytoplasmic genetics of mammalian cells: conditional sensitivity to mitochondrial inhibitors and isolation of new mutant phenotypes. *Somat. Cell Genet.* 5 833–845. 10.1007/bf01542645 296586

[B67] Kaelin W. G. Jr. McKnight S. L. (2013). Influence of metabolism on epigenetics and disease. *Cell* 153 56–69. 10.1016/j.cell.2013.03.004 23540690 PMC3775362

[B68] Kang P. J. Zheng J. Lee G. Son D. Kim I. Y. Song G. (2019). Glycine decarboxylase regulates the maintenance and induction of pluripotency via metabolic control. *Metab. Eng.* 53 35–47. 10.1016/j.ymben.2019.02.003 30779965

[B69] Khacho M. Clark A. Svoboda D. S. Azzi J. MacLaurin J. G. Meghaizel C. (2016). Mitochondrial dynamics impacts stem cell identity and fate decisions by regulating a nuclear transcriptional program. *Cell Stem Cell* 19 232–247. 10.1016/j.stem.2016.04.015 27237737

[B70] Kida Y. S. Kawamura T. Wei Z. Sogo T. Jacinto S. Shigeno A. (2015). ERRs mediate a metabolic switch required for somatic cell reprogramming to pluripotency. *Cell Stem Cell* 16 547–555. 10.1016/j.stem.2015.03.001 25865501 PMC4427539

[B71] Kilens S. Meistermann D. Moreno D. Chariau C. Gaignerie A. Reignier A. (2018). Parallel derivation of isogenic human primed and naive induced pluripotent stem cells. *Nat. Commun.* 9:360. 10.1038/s41467-017-02107-w 29367672 PMC5783949

[B72] Kim H. Jang H. Kim T. W. Kang B.-H. Lee S. E. Jeon Y. K. (2015). Core pluripotency factors directly regulate metabolism in embryonic stem cell to maintain pluripotency. *Stem Cell* 33 2699–2711. 10.1002/stem.2073 26059508

[B73] Kim K. Doi A. Wen B. Ng K. Zhao R. Cahan P. (2010). Epigenetic memory in induced pluripotent stem cells. *Nature* 467 285–290. 10.1038/nature09342 20644535 PMC3150836

[B74] Kime C. Sakaki-Yumoto M. Goodrich L. Hayashi Y. Sami S. Derynck R. (2016). Autotaxin-mediated lipid signaling intersects with LIF and BMP signaling to promote the naive pluripotency transcription factor program. *Proc. Natl. Acad. Sci. U.S.A.* 113 12478–12483. 10.1073/pnas.1608564113 27738243 PMC5098653

[B75] Knobloch M. Braun S. M. Zurkirchen L. von Schoultz C. Zamboni N. Arauzo-Bravo M. J. (2013). Metabolic control of adult neural stem cell activity by Fasn-dependent lipogenesis. *Nature* 493 226–230. 10.1038/nature11689 23201681 PMC3587167

[B76] Kondoh H. Lleonart M. E. Nakashima Y. Yokode M. Tanaka M. Bernard D. (2007). A high glycolytic flux supports the proliferative potential of murine embryonic stem cells. *Antioxid. Redox. Signal.* 9 293–299. 10.1089/ars.2007.9.ft-14 17184172

[B77] Labbe K. Murley A. Nunnari J. (2014). Determinants and functions of mitochondrial behavior. *Annu. Rev. Cell Dev. Biol.* 30 357–391. 10.1146/annurev-cellbio-101011-155756 25288115

[B78] Lee M. R. Mantel C. Lee S. A. Moon S. H. Broxmeyer H. E. (2016). MiR-31/SDHA axis regulates reprogramming efficiency through mitochondrial metabolism. *Stem Cell Rep.* 7 1–10. 10.1016/j.stemcr.2016.05.012 27346679 PMC4944586

[B79] Lee M. R. Prasain N. Chae H. D. Kim Y. J. Mantel C. Yoder M. C. (2013). Epigenetic regulation of NANOG by miR-302 cluster-MBD2 completes induced pluripotent stem cell reprogramming. *Stem Cells* 31 666–681. 10.1002/stem.1302 23255147 PMC3904370

[B80] Lee Y. L. Peng Q. Fong S. W. Chen A. C. Lee K. F. Ng E. H. (2012). Sirtuin 1 facilitates generation of induced pluripotent stem cells from mouse embryonic fibroblasts through the miR-34a and p53 pathways. *PLoS One* 7:e45633. 10.1371/journal.pone.0045633 23029150 PMC3448677

[B81] Lees J. G. Cliff T. S. Gammilonghi A. Ryall J. G. Dalton S. Gardner D. K. (2019). Oxygen regulates human pluripotent stem cell metabolic flux. *Stem Cells Int.* 2019:8195614. 10.1155/2019/8195614 31236115 PMC6545818

[B82] Lees J. G. Rathjen J. Sheedy J. R. Gardner D. K. Harvey A. J. (2015). Distinct profiles of human embryonic stem cell metabolism and mitochondria identified by oxygen. *Reproduction* 150 367–382. 10.1530/REP-14-0633 26159831

[B83] Lengner C. J. Gimelbrant A. A. Erwin J. A. Cheng A. W. Guenther M. G. Welstead G. G. (2010). Derivation of Pre-X inactivation human embryonic stem cells under physiological oxygen concentrations. *Cell* 141 872–883. 10.1016/j.cell.2010.04.010 20471072

[B84] Liesa M. Shirihai O. S. (2013). Mitochondrial dynamics in the regulation of nutrient utilization and energy expenditure. *Cell Metab.* 17 491–506. 10.1016/j.cmet.2013.03.002 23562075 PMC5967396

[B85] Lin Z. Liu F. Shi P. Song A. Huang Z. Zou D. (2018). Fatty acid oxidation promotes reprogramming by enhancing oxidative phosphorylation and inhibiting protein kinase C. *Stem Cell Res. Ther.* 9:47. 10.1186/s13287-018-0792-6 29482657 PMC5937047

[B86] Locasale J. W. (2013). Serine, glycine and one-carbon units: cancer metabolism in full circle. *Nat. Rev. Cancer* 13 572–583. 10.1038/nrc3557 23822983 PMC3806315

[B87] Lonergan T. Bavister B. Brenner C. (2007). Mitochondria in stem cells. *Mitochondrion* 7 289–296. 10.1016/j.mito.2007.05.002 17588828 PMC3089799

[B88] Lonergan T. Brenner C. Bavister B. (2006). Differentiation-related changes in mitochondrial properties as indicators of stem cell competence. *J. Cell Physiol.* 208 149–153. 10.1002/jcp.20641 16575916

[B89] Lunt S. Y. Vander Heiden M. G. (2011). Aerobic glycolysis: meeting the metabolic requirements of cell proliferation. *Ann. Rev. Cell Dev. Biol.* 27 441–464. 10.1146/annurev-cellbio-092910-154237 21985671

[B90] Ma H. Folmes C. D. Wu J. Morey R. Mora-Castilla S. Ocampo A. (2015a). Metabolic rescue in pluripotent cells from patients with mtDNA disease. *Nature* 524 234–238. 10.1038/nature14546 26176921

[B91] Ma T. Li J. Xu Y. Yu C. Xu T. Wang H. (2015b). Atg5-independent autophagy regulates mitochondrial clearance and is essential for iPSC reprogramming. *Nat. Cell Biol.* 17 1379–1387. 10.1038/ncb3256 26502054

[B92] Mah N. Wang Y. Liao M. C. Prigione A. Jozefczuk J. Lichtner B. (2011). Molecular insights into reprogramming-initiation events mediated by the OSKM gene regulatory network. *PLoS One* 6:e24351. 10.1371/journal.pone.0024351 21909390 PMC3164204

[B93] Mathieu J. Ruohola-Baker H. (2017). Metabolic remodeling during the loss and acquisition of pluripotency. *Development* 144 541–551. 10.1242/dev.128389 28196802 PMC5312031

[B94] Mathieu J. Zhang Z. Nelson A. Lamba D. A. Reh T. A. Ware C. (2013). Hypoxia induces re-entry of committed cells into pluripotency. *Stem Cells* 31 1737–1748. 10.1002/stem.1446 23765801 PMC3921075

[B95] Mathieu J. Zhou W. Xing Y. Sperber H. Ferreccio A. Agoston Z. (2014). Hypoxia-inducible factors have distinct and stage-specific roles during reprogramming of human cells to pluripotency. *Cell Stem Cell* 14 592–605. 10.1016/j.stem.2014.02.012 24656769 PMC4028142

[B96] Matilainen O. Quiros P. M. Auwerx J. (2017). Mitochondria and epigenetics - crosstalk in homeostasis and stress. *Trend Cell Biol.* 27 453–463. 10.1016/j.tcb.2017.02.004 28274652

[B97] Mattenberger Y. James D. I. Martinou J. C. (2003). Fusion of mitochondria in mammalian cells is dependent on the mitochondrial inner membrane potential and independent of microtubules or actin. *FEBS Lett.* 538 53–59. 10.1016/s0014-5793(03)00124-8 12633852

[B98] Meissen J. K. Yuen B. T. K. Kind T. Riggs J. W. Barupal D. K. Knoepfler P. S. (2012). Induced pluripotent stem cells show metabolomic differences to embryonic stem cells in polyunsaturated phosphatidylcholines and primary metabolism. *PLoS One* 7:e46770. 10.1371/journal.pone.0046770 23077522 PMC3471894

[B99] Mohyeldin A. Garzon-Muvdi T. Quinones-Hinojosa A. (2010). Oxygen in stem cell biology: a critical component of the stem cell niche. *Cell Stem Cell* 7 150–161. 10.1016/j.stem.2010.07.007 20682444

[B100] Moussaieff A. Rouleau M. Kitsberg D. Cohen M. Levy G. Barasch D. (2015). Glycolysis-mediated changes in acetyl-CoA and histone acetylation control the early differentiation of embryonic stem cells. *Cell Metab.* 21 392–402. 10.1016/j.cmet.2015.02.002 25738455

[B101] Mu W. L. Wang Y. J. Xu P. Hao D. L. Liu X. Z. Wang T. T. (2015). Sox2 deacetylation by sirt1 is involved in mouse somatic reprogramming. *Stem Cells* 33 2135–2147. 10.1002/stem.2012 25940188

[B102] Nichols J. Smith A. (2009). Naive and primed pluripotent states. *Cell Stem Cell* 4 487–492. 10.1016/j.stem.2009.05.015 19497275

[B103] Oey N. A. den Boer M. E. Wijburg F. A. Vekemans M. Auge J. Steiner C. (2005). Long-chain fatty acid oxidation during early human development. *Pediatr. Res.* 57 755–759. 10.1203/01.PDR.0000161413.42874.74 15845636

[B104] Ohi Y. Qin H. Hong C. Blouin L. Polo J. M. Guo T. (2011). Incomplete DNA methylation underlies a transcriptional memory of somatic cells in human iPS cells. *Nat. Cell Biol.* 13 541–549. 10.1038/ncb2239 21499256 PMC3987913

[B105] O’Reilly C. Qi Q. Peters J. L. Cheng Y. Yoon S. O. Han M. J. (2019). The primitive growth factor NME7AB induces mitochondrially active naive-like pluripotent stem cells. *Biochem. Biophys. Rep.* 20:100656. 10.1016/j.bbrep.2019.100656 31467990 PMC6711853

[B106] Osorno R. Tsakiridis A. Wong F. Cambray N. Economou C. Wilkie R. (2012). The developmental dismantling of pluripotency is reversed by ectopic Oct4 expression. *Development* 139 2288–2298. 10.1242/dev.078071 22669820 PMC3367440

[B107] Panopoulos A. D. Yanes O. Ruiz S. Kida Y. S. Diep D. Tautenhahn R. (2012). The metabolome of induced pluripotent stem cells reveals metabolic changes occurring in somatic cell reprogramming. *Cell Res.* 22 168–177. 10.1038/cr.2011.177 22064701 PMC3252494

[B108] Pantaleon M. Kaye P. L. (1998). Glucose transporters in preimplantation development. *Rev. Reprod.* 3 77–81. 10.1530/ror.0.0030077 9685185

[B109] Park S. J. Lee S. A. Prasain N. Bae D. Kang H. Ha T. (2017). Metabolome profiling of partial and fully reprogrammed induced pluripotent stem cells. *Stem Cells Dev.* 26 734–742. 10.1089/scd.2016.0320 28346802

[B110] Perales-Clemente E. Folmes C. D. Terzic A. (2014). Metabolic regulation of redox status in stem cells. *Antioxid. Redox. Signal.* 21 1648–1659. 10.1089/ars.2014.6000 24949895 PMC4174422

[B111] Prasad S. M. Czepiel M. Cetinkaya C. Smigielska K. Weli S. C. Lysdahl H. (2009). Continuous hypoxic culturing maintains activation of notch and allows long-term propagation of human embryonic stem cells without spontaneous differentiation. *Cell Prolif.* 42 63–74. 10.1111/j.1365-2184.2008.00571.x 19143764 PMC6496631

[B112] Prigione A. Hossini A. M. Lichtner B. Serin A. Fauler B. Megges M. (2011). Mitochondrial-associated cell death mechanisms are reset to an embryonic-like state in aged donor-derived iPS cells harboring chromosomal aberrations. *PLoS One* 6:e27352. 10.1371/journal.pone.0027352 22110631 PMC3215709

[B113] Prigione A. Rohwer N. Hoffmann S. Mlody B. Drews K. Bukowiecki R. (2014). HIF1alpha modulates cell fate reprogramming through early glycolytic shift and upregulation of PDK1-3 and PKM2. *Stem Cells* 32 364–376. 10.1002/stem.1552 24123565 PMC5730046

[B114] Qin S. T. Yang D. L. Chen K. Li H. L. Zhang L. Q. Li Y. (2017). Pkm2 can enhance pluripotency in ESCs and promote somatic cell reprogramming to iPSCs. *Oncotarget* 8 84276–84284. 10.18632/oncotarget.20685 29137422 PMC5663594

[B115] Rizzuto R. Brini M. Murgia M. Pozzan T. (1993). Microdomains with high Ca2+ close to IP3-sensitive channels that are sensed by neighboring mitochondria. *Science* 262 744–747. 10.1126/science.8235595 8235595

[B116] Ryall J. G. Cliff T. Dalton S. Sartorelli V. (2015). Metabolic Reprogramming of Stem Cell Epigenetics. *Cell Stem Cell* 17 651–662. 10.1016/j.stem.2015.11.012 26637942 PMC4672395

[B117] Ryu J. M. Han H. J. (2011). L-threonine regulates G1/S phase transition of mouse embryonic stem cells via PI3K/Akt, MAPKs, and mTORC pathways. *J. Biol. Chem.* 286 23667–23678. 10.1074/jbc.M110.216283 21550972 PMC3129147

[B118] Schell J. C. Wisidagama D. R. Bensard C. Zhao H. Wei P. Tanner J. (2017). Control of intestinal stem cell function and proliferation by mitochondrial pyruvate metabolism. *Nat. Cell Biol.* 19 1027–1036. 10.1038/ncb3593 28812582 PMC6137334

[B119] Schieke S. M. Ma M. Cao L. McCoy J. P. Jr. Liu C. Hensel N. F. (2008). Mitochondrial metabolism modulates differentiation and teratoma formation capacity in mouse embryonic stem cells. *J. Biol. Chem.* 283 28506–28512. 10.1074/jbc.M802763200 18713735 PMC2568919

[B120] Seo B. J. Yoon S. H. Do J. T. (2018). Mitochondrial dynamics in stem cells and differentiation. *Int. J. Mol. Sci.* 19:3893. 10.3390/ijms19123893 30563106 PMC6321186

[B121] Shiraki N. Shiraki Y. Tsuyama T. Obata F. Miura M. Nagae G. (2014). Methionine metabolism regulates maintenance and differentiation of human pluripotent stem cells. *Cell Metab.* 19 780–794. 10.1016/j.cmet.2014.03.017 24746804

[B122] Shyh-Chang N. Daley G. Q. Cantley L. C. (2013a). Stem cell metabolism in tissue development and aging. *Development* 140 2535–2547. 10.1242/dev.091777 23715547 PMC3666381

[B123] Shyh-Chang N. Locasale J. W. Lyssiotis C. A. Zheng Y. Teo R. Y. Ratanasirintrawoot S. (2013b). Influence of threonine metabolism on S-adenosylmethionine and histone methylation. *Science* 339 222–226. 10.1126/science.1226603 23118012 PMC3652341

[B124] Shyh-Chang N. Zhu H. Yvanka de Soysa T. Shinoda G. Seligson M. T. Tsanov K. M. (2013c). Lin28 enhances tissue repair by reprogramming cellular metabolism. *Cell* 155 778–792. 10.1016/j.cell.2013.09.059 24209617 PMC3917449

[B125] Shyh-Chang N. Zheng Y. Locasale J. W. Cantley L. C. (2011). Human pluripotent stem cells decouple respiration from energy production. *EMBO J.* 30 4851–4852. 10.1038/emboj.2011.436 22166995 PMC3242981

[B126] Si X. Chen W. Guo X. Chen L. Wang G. Xu Y. (2013). Activation of GSK3beta by Sirt2 is required for early lineage commitment of mouse embryonic stem cell. *PLoS One* 8:e76699. 10.1371/journal.pone.0076699 24204656 PMC3800056

[B127] Simsek T. Kocabas F. Zheng J. Deberardinis R. J. Mahmoud A. I. Olson E. N. (2010). The distinct metabolic profile of hematopoietic stem cells reflects their location in a hypoxic niche. *Cell Stem Cell* 7 380–390. 10.1016/j.stem.2010.07.011 20804973 PMC4159713

[B128] Son M. J. Kwon Y. Son M. Y. Seol B. Choi H. S. Ryu S. W. (2015). Mitofusins deficiency elicits mitochondrial metabolic reprogramming to pluripotency. *Cell Death Differ.* 22 1957–1969. 10.1038/cdd.2015.43 25882047 PMC4816104

[B129] Sone M. Morone N. Nakamura T. Tanaka A. Okita K. Woltjen K. (2017). Hybrid cellular metabolism coordinated by Zic3 and Esrrb synergistically enhances induction of naive pluripotency. *Cell Metab.* 25 1103.e–1117.e. 10.1016/j.cmet.2017.04.017 28467928

[B130] Song C. Xu F. Ren Z. Zhang Y. Meng Y. Yang Y. (2019). Elevated exogenous pyruvate potentiates mesodermal differentiation through metabolic modulation and AMPK/mTOR pathway in human embryonic stem cells. *Stem Cell Rep.* 13 338–351. 10.1016/j.stemcr.2019.06.003 31353224 PMC6700476

[B131] Sperber H. Mathieu J. Wang Y. Ferreccio A. Hesson J. Xu Z. (2015). The metabolome regulates the epigenetic landscape during naive-to-primed human embryonic stem cell transition. *Nat. Cell Biol.* 17 1523–1535. 10.1038/ncb3264 26571212 PMC4662931

[B132] Spyrou J. Gardner D. K. Harvey A. J. (2019). Metabolomic and transcriptional analyses reveal atmospheric oxygen during human induced pluripotent stem cell generation impairs metabolic reprogramming. *Stem Cells* 37 1042–1056. 10.1002/stem.3029 31042329

[B133] St John J. C. (2016). Mitochondrial DNA copy number and replication in reprogramming and differentiation. *Semin. Cell Dev. Biol.* 52 93–101. 10.1016/j.semcdb.2016.01.028 26827792

[B134] St John J. C. Ramalho-Santos J. Gray H. L. Petrosko P. Rawe V. Y. Navara C. S. (2005). The expression of mitochondrial DNA transcription factors during early cardiomyocyte in vitro differentiation from human embryonic stem cells. *Clon. Stem Cell* 7 141–153. 10.1089/clo.2005.7.141 16176124

[B135] Suhr S. T. Chang E. A. Tjong J. Alcasid N. Perkins G. A. Goissis M. D. (2010). Mitochondrial rejuvenation after induced pluripotency. *PLoS One* 5:e14095. 10.1371/journal.pone.0014095 21124794 PMC2991355

[B136] Takahashi K. Tanabe K. Ohnuki M. Narita M. Ichisaka T. Tomoda K. (2007). Induction of Pluripotent stem cells from adult human fibroblasts by defined factors. *Cell* 131 861–872. 10.1016/j.cell.2007.11.019 18035408

[B137] Takahashi K. Yamanaka S. (2006). Induction of pluripotent stem cells from mouse embryonic and adult fibroblast cultures by defined factors. *Cell* 126 663–676. 10.1016/j.cell.2006.07.024 16904174

[B138] Takashima Y. Guo G. Loos R. Nichols J. Ficz G. Krueger F. (2014). Resetting transcription factor control circuitry toward ground-state pluripotency in human. *Cell* 158 1254–1269. 10.1016/j.cell.2014.08.029 25215486 PMC4162745

[B139] Takubo K. Nagamatsu G. Kobayashi C. I. Nakamura-Ishizu A. Kobayashi H. Ikeda E. (2013). Regulation of glycolysis by Pdk functions as a metabolic checkpoint for cell cycle quiescence in hematopoietic stem cells. *Cell Stem Cell* 12 49–61. 10.1016/j.stem.2012.10.011 23290136 PMC6592822

[B140] Tang S. Fang Y. Huang G. Xu X. Padilla-Banks E. Fan W. (2017). Methionine metabolism is essential for SIRT1-regulated mouse embryonic stem cell maintenance and embryonic development. *EMBO J.* 36 3175–3193. 10.15252/embj.201796708 29021282 PMC5666621

[B141] Tesar P. J. Chenoweth J. G. Brook F. A. Davies T. J. Evans E. P. Mack D. L. (2007). New cell lines from mouse epiblast share defining features with human embryonic stem cells. *Nature* 448 196–199. 10.1038/nature05972 17597760

[B142] TeSlaa T. Chaikovsky A. C. Lipchina I. Escobar S. L. Hochedlinger K. Huang J. (2016). alpha-Ketoglutarate accelerates the initial differentiation of primed human pluripotent stem cells. *Cell Metab.* 24 485–493. 10.1016/j.cmet.2016.07.002 27476976 PMC5023506

[B143] Teslaa T. Teitell M. A. (2015). Pluripotent stem cell energy metabolism: an update. *EMBO J.* 34 138–153. 10.15252/embj.201490446 25476451 PMC4337063

[B144] Theunissen T. W. Powell B. E. Wang H. Mitalipova M. Faddah D. A. Reddy J. (2014). Systematic identification of culture conditions for induction and maintenance of naive human pluripotency. *Cell Stem Cell* 15 471–487. 10.1016/j.stem.2014.07.002 25090446 PMC4184977

[B145] Thomson J. A. Itskovitz-Eldor J. Shapiro S. S. Waknitz M. A. Swiergiel J. J. Marshall V. S. (1998). Embryonic stem cell lines derived from human blastocysts. *Science* 282 1145–1147. 10.1126/science.282.5391.1145 9804556

[B146] Tischler J. Gruhn W. H. Reid J. Allgeyer E. Buettner F. Marr C. (2019). Metabolic regulation of pluripotency and germ cell fate through alpha-ketoglutarate. *EMBO J.* 38:e99518. 10.15252/embj.201899518 30257965 PMC6315289

[B147] Tohyama S. Fujita J. Hishiki T. Matsuura T. Hattori F. Ohno R. (2016). Glutamine oxidation is indispensable for survival of human pluripotent stem cells. *Cell Metab.* 23 663–674. 10.1016/j.cmet.2016.03.001 27050306

[B148] Turner J. Quek L.-E. Titmarsh D. Krömer J. O. Kao L.-P. Nielsen L. (2014). Metabolic profiling and flux analysis of MEL-2 human embryonic stem cells during exponential growth at physiological and atmospheric oxygen concentrations. *PLoS One* 9:e112757. 10.1371/journal.pone.0112757 25412279 PMC4239018

[B149] Vander Heiden M. G. Cantley L. C. Thompson C. B. (2009). Understanding the warburg effect: the metabolic requirements of cell proliferation. *Science* 324 1029–1033. 10.1126/science.1160809 19460998 PMC2849637

[B150] Vardhana S. A. Arnold P. K. Rosen B. P. Chen Y. Carey B. W. Huangfu D. (2019). Glutamine independence is a selectable feature of pluripotent stem cells. *Nat. Metab.* 1 676–687. 10.1038/s42255-019-0082-3 31511848 PMC6737941

[B151] Varum S. Momčilović O. Castro C. Ben-Yehudah A. Ramalho-Santos J. Navara C. S. (2009). Enhancement of human embryonic stem cell pluripotency through inhibition of the mitochondrial respiratory chain. *Stem Cell Res.* 3 142–156. 10.1016/j.scr.2009.07.002 19716358 PMC2837416

[B152] Varum S. Rodrigues A. S. Moura M. B. Momcilovic O. Easley C. A. Ramalho-Santos J. (2011). Energy metabolism in human pluripotent stem cells and their differentiated counterparts. *PLoS One* 6:e20914. 10.1371/journal.pone.0020914 21698063 PMC3117868

[B153] Vazquez-Martin A. Cufi S. Corominas-Faja B. Oliveras-Ferraros C. Vellon L. Menendez J. A. (2012). Mitochondrial fusion by pharmacological manipulation impedes somatic cell reprogramming to pluripotency: new insight into the role of mitophagy in cell stemness. *Aging* 4 393–401. 10.18632/aging.100465 22713507 PMC3409676

[B154] Vernardis S. I. Terzoudis K. Panoskaltsis N. Mantalaris A. (2017). Human embryonic and induced pluripotent stem cells maintain phenotype but alter their metabolism after exposure to ROCK inhibitor. *Sci. Rep.* 7 1–11. 10.1038/srep42138 28165055 PMC5292706

[B155] Wang J. Alexander P. McKnight S. L. (2011). Metabolic specialization of mouse embryonic stem cells. *Cold Spring Harb. Symp. Quant. Biol.* 76 183–193. 10.1101/sqb.2011.76.010835 22071264

[B156] Wang J. Alexander P. Wu L. Hammer R. Cleaver O. McKnight S. L. (2009). Dependence of mouse embryonic stem cells on threonine catabolism. *Science* 325 435–439. 10.1126/science.1173288 19589965 PMC4373593

[B157] Wang L. Zhang T. Wang L. Cai Y. Zhong X. He X. (2017). Fatty acid synthesis is critical for stem cell pluripotency via promoting mitochondrial fission. *EMBO J.* 36 1330–1347. 10.15252/embj.201695417 28377463 PMC5430220

[B158] Ware C. B. Nelson A. M. Mecham B. Hesson J. Zhou W. Jonlin E. C. (2014). Derivation of naive human embryonic stem cells. *Proc. Natl. Acad. Sci. U.S.A.* 111 4484–4489. 10.1073/pnas.1319738111 24623855 PMC3970494

[B159] Washington J. M. Rathjen J. Felquer F. Lonic A. Bettess M. D. Hamra N. (2010). L-Proline induces differentiation of ES cells: a novel role for an amino acid in the regulation of pluripotent cells in culture. *Am. J. Physiol. Cell Physiol.* 298 C982–C992. 10.1152/ajpcell.00498.2009 20164384

[B160] Weinberger L. Ayyash M. Novershtern N. Hanna J. H. (2016). Dynamic stem cell states: naive to primed pluripotency in rodents and humans. *Nat. Rev. Mol. Cell Biol.* 17 155–169. 10.1038/nrm.2015.28 26860365

[B161] Wellen K. E. Hatzivassiliou G. Sachdeva U. M. Bui T. V. Cross J. R. Thompson C. B. (2009). ATP-citrate lyase links cellular metabolism to histone acetylation. *Science* 324 1076–1080. 10.1126/science.1164097 19461003 PMC2746744

[B162] Wellen K. E. Thompson C. B. (2012). A two-way street: reciprocal regulation of metabolism and signalling. *Nat. Rev. Mol. Cell Biol.* 13 270–276. 10.1038/nrm3305 22395772

[B163] Wheaton W. W. Weinberg S. E. Hamanaka R. B. Soberanes S. Sullivan L. B. Anso E. (2014). Metformin inhibits mitochondrial complex I of cancer cells to reduce tumorigenesis. *eLife* 3:e02242. 10.7554/eLife.02242 24843020 PMC4017650

[B164] Ying Q. L. Wray J. Nichols J. Batlle-Morera L. Doble B. Woodgett J. (2008). The ground state of embryonic stem cell self-renewal. *Nature* 453 519–523. 10.1038/nature06968 18497825 PMC5328678

[B165] Yoshida Y. Takahashi K. Okita K. Ichisaka T. Yamanaka S. (2009). Hypoxia enhances the generation of induced pluripotent stem cells. *Cell Stem Cell* 5 237–241. 10.1016/j.stem.2009.08.001 19716359

[B166] Yu L. Ji K. Y. Zhang J. Xu Y. Ying Y. Mai T. (2019). Core pluripotency factors promote glycolysis of human embryonic stem cells by activating GLUT1 enhancer. *Protein Cell* 10 668–680. 10.1007/s13238-019-0637-9 31152430 PMC6711954

[B167] Zhang H. Badur M. G. Divakaruni A. S. Parker S. J. Jager C. Hiller K. (2016a). Distinct metabolic states can support self-renewal and lipogenesis in human pluripotent stem cells under different culture conditions. *Cell Rep.* 16 1536–1547. 10.1016/j.celrep.2016.06.102 27477285 PMC4981511

[B168] Zhang J. Khvorostov I. Hong J. S. Oktay Y. Vergnes L. Nuebel E. (2011). UCP2 regulates energy metabolism and differentiation potential of human pluripotent stem cells. *EMBO J.* 30 4860–4873. 10.1038/emboj.2011.401 22085932 PMC3243621

[B169] Zhang J. Nuebel E. Daley G. Q. Koehler C. M. Teitell M. A. (2012). Metabolic regulation in pluripotent stem cells during reprogramming and self-renewal. *Cell Stem Cell* 11 589–595. 10.1016/j.stem.2012.10.005 23122286 PMC3492890

[B170] Zhang J. Ratanasirintrawoot S. Chandrasekaran S. Wu Z. Ficarro S. B. Yu C. (2016b). LIN28 regulates stem cell metabolism and conversion to primed pluripotency. *Cell Stem Cell* 19 66–80. 10.1016/j.stem.2016.05.009 27320042 PMC13373712

[B171] Zhang J. Zhao J. Dahan P. Lu V. Zhang C. Li H. (2018). Metabolism in pluripotent stem cells and early mammalian development. *Cell Metab.* 27 332–338. 10.1016/j.cmet.2018.01.008 29414683

[B172] Zhong X. Cui P. Cai Y. Wang L. He X. Long P. (2019). Mitochondrial dynamics is critical for the full pluripotency and embryonic developmental potential of pluripotent stem cells. *Cell Metab.* 29 979–992.e4. 10.1016/j.cmet.2018.11.007 30527743

[B173] Zhou W. Choi M. Margineantu D. Margaretha L. Hesson J. Cavanaugh C. (2012). HIF1alpha induced switch from bivalent to exclusively glycolytic metabolism during ESC-to-EpiSC/hESC transition. *EMBO J.* 31 2103–2116. 10.1038/emboj.2012.71 22446391 PMC3343469

[B174] Zhu S. Li W. Zhou H. Wei W. Ambasudhan R. Lin T. (2010). Reprogramming of human primary somatic cells by OCT4 and chemical compounds. *Cell Stem Cell* 7 651–655. 10.1016/j.stem.2010.11.015 21112560 PMC3812930

